# RHINO: An Integrative Multi‐Omics Framework Linking Circadian Physiology to Precision Medicine

**DOI:** 10.1002/advs.76371

**Published:** 2026-07-07

**Authors:** Ying Chen, Chengxuan Chen, Dishu Zhou, Panpan Liu, Roberto E. López‐Valiente, Yuan Liu, Cam Mong La, Isabella Beraldo Xavier, Sean M. Hartig, Pradip Saha, Zheng Sun, Leng Han, Dongyin Guan

**Affiliations:** ^1^ Division of Diabetes Endocrinology, and Metabolism Department of Medicine Baylor College of Medicine Houston Texas USA; ^2^ Department of Biostatistics and Health Data Science Indiana University School of Medicine Indianapolis Indiana USA

**Keywords:** circadian medicine, circadian rhythm, data integration, ERα, multi‐omics, RHINO

## Abstract

Circadian rhythms are involved in nearly all physiological processes, and their disruption increases disease risk. Aligning drug administration timing with the body's internal clock, known as circadian medicine, holds promise for improving treatment efficacy and safety. However, systematic strategies to identify circadian‐regulated therapeutic targets remain limited. Here, we develop RHINO (RHythmic Interacting Network for multi‐Omics), an integrative multi‐omics framework for exploring circadian regulation across diverse genetic and disease contexts, and prioritizing druggable circadian targets. Applying RHINO reveals that human genetic variation broadly shapes rhythmic gene expression, with approximately 80% of FDA‐approved drug targets exhibiting genotype‐dependent rhythmicity, thereby substantially expanding the druggable target landscape for more precise circadian medicine. Circadian analyses across cancer types and diseases further identify genes that consistently lose rhythmic expression, highlighting conserved circadian vulnerabilities linked to disease progression. Integrative regulatory analyses nominate upstream transcriptional regulators, including Estrogen Receptor α, associated with rhythmic disruption. Although the circadian rhythm of estrogen signaling has been recognized for decades, we show that this rhythmicity exhibits time‐of‐day–dependent metabolic responses in preclinical mouse models. Together, RHINO provides a community‐accessible multi‐omics platform that integrates genetic variation, disease‐associated circadian remodeling, and regulatory inference to advance circadian medicine (https://hanlaboratory.com/RHINO).

## Graphical abstract

RHINO (RHythmic Interacting Network for multi‐Omics) is an integrative framework that maps circadian regulation across diverse genetic and disease contexts and prioritizes druggable circadian targets. Released as an AI‐powered interactive web portal, RHINO unifies genetic, regulatory, disease, and drug–target information, enabling context‐specific analyses of circadian biology and guiding the discovery of time‐dependent therapeutics (https://hanlaboratory.com/RHINO).

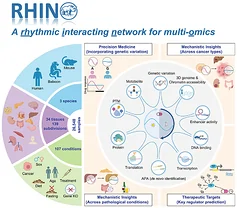

## 1 Introduction

Circadian rhythms are endogenous timing systems that allow organisms to anticipate and adapt to environmental changes following a 24‐h cycle [1]. These rhythms are influenced by various environmental and metabolic stimuli, such as light and food intake, and coordinate diverse biological processes, including energy metabolism, hormone secretion, immune responses, and cell proliferation [2]. Epidemiological studies suggest that circadian misalignment, caused by sleep deprivation, irregular eating, or chronic jet lag, is associated with increased risks of a wide range of diseases, including obesity, diabetes, and cancer [3, 4]. Conversely, aligning external environmental cues with internal circadian rhythms, one of the major goals of circadian medicine, has yielded beneficial outcomes across cancer, metabolic, cardiovascular, neurological, and age‐related diseases [3]. The growing understanding of circadian rhythms and their impact on physiology and disease has positioned circadian medicine as a promising and innovative therapeutic approach [3].

To translate circadian physiology into clinical applications, therapeutic strategies generally fall into three categories. The first is to “train the clock”, i.e., interventions that enhance or maintain robust circadian rhythms in feeding/fasting, sleep/wake, or light/dark cycles. For example, aligning mealtimes with the physically active phase has been shown to improve overall metabolic health, particularly by managing blood glucose levels and preventing the development of type 2 diabetes [5, 6]. The second is to “drug the clock”, i.e., using small‐molecule agents to target a circadian clock molecule. For example, both in vitro and in vivo studies demonstrated that REV‐ERB agonists inhibit autophagy and *de novo* lipogenesis, producing selective anticancer effects across multiple cancer types, including brain, leukemia, breast, colon, and melanoma cancers [7, 8]. The third is to “clock the drugs”, i.e., optimizing the timing of drug administration to improve efficacy and reduce side effects. This strategy is also known as chronotherapy. Since many anticancer drugs have cytotoxic effects on both malignant and healthy cells, a major goal of chronotherapeutic studies for cancer is to reduce host toxicity. For example, in advanced ovarian cancer, administering doxorubicin in the morning and cisplatin in the evening reduced complications and renal toxicity compared with administering them at opposite times of day [9, 10]. Together, these strategies underscore the therapeutic potential of circadian medicine. However, broader clinical implementation requires comprehensive identification of circadian‐regulated targets and precise optimization of drug administration timing.

To address this gap, we constructed a RHythmic Interacting Network for multi‐Omics (RHINO) framework by integrating genetic, epigenetic, transcriptional, and post‐transcriptional regulatory information related to circadian rhythms and incorporating the identified circadian genes with drug target annotation. This framework enables integration of genetic variation with rhythmic gene expression and mapping of genetically associated rhythmic genes to approved drugs, revealing that approximately 80% of FDA‐approved drug targets exhibit rhythmic expression, compared with the previously reported 56% [11]. This increase expands the scope of druggable targets for circadian medicine and reveals new opportunities for time‐optimized therapeutic strategies. Beyond genetic regulation, we systematically characterize circadian remodeling of rhythmic genes across diverse human cancers, leveraging cancer genomic databases as the most comprehensive multi‐tissue transcriptomic resource currently available for population‐level circadian analysis, identifying conserved circadian vulnerabilities across pathological states and temporally regulated targets with clinical relevance. Comparative analyses across multiple cancer types and additional pathological conditions in mouse models further revealed genes that consistently lose rhythmic expression, indicating conserved circadian disruption across disease states. To uncover the regulatory mechanisms underlying these alterations, integrative multi‐omics analyses incorporating epigenomic data identified upstream regulators of rhythmic transcription, enabling prioritization of candidate regulators for therapeutic intervention. For example, although the circadian rhythms of circulating estrogen have been reported for decades, the functional significance of this rhythmicity is still unclear.

Here, we identify estrogen receptor α (ERα) as one of the top upstream regulators for genes that consistently lose rhythmicity and show that metabolic responses to estrogen exhibit time‐of‐day specificity in preclinical models, supporting the functional relevance of circulating estrogen rhythmicity. To support systematic exploration of circadian regulation and chronotherapeutic strategies, RHINO was released as an AI‐powered interactive web portal that integrates genetic, regulatory, disease, and drug–target information, enabling context‐specific analyses of circadian regulation and exploration of time‐dependent therapeutics.

## 2 Results

### 2.1 Human Genetic Variation Expands the Potential Therapeutic Targets for Precision Circadian Medicine

**FIGURE 1:**
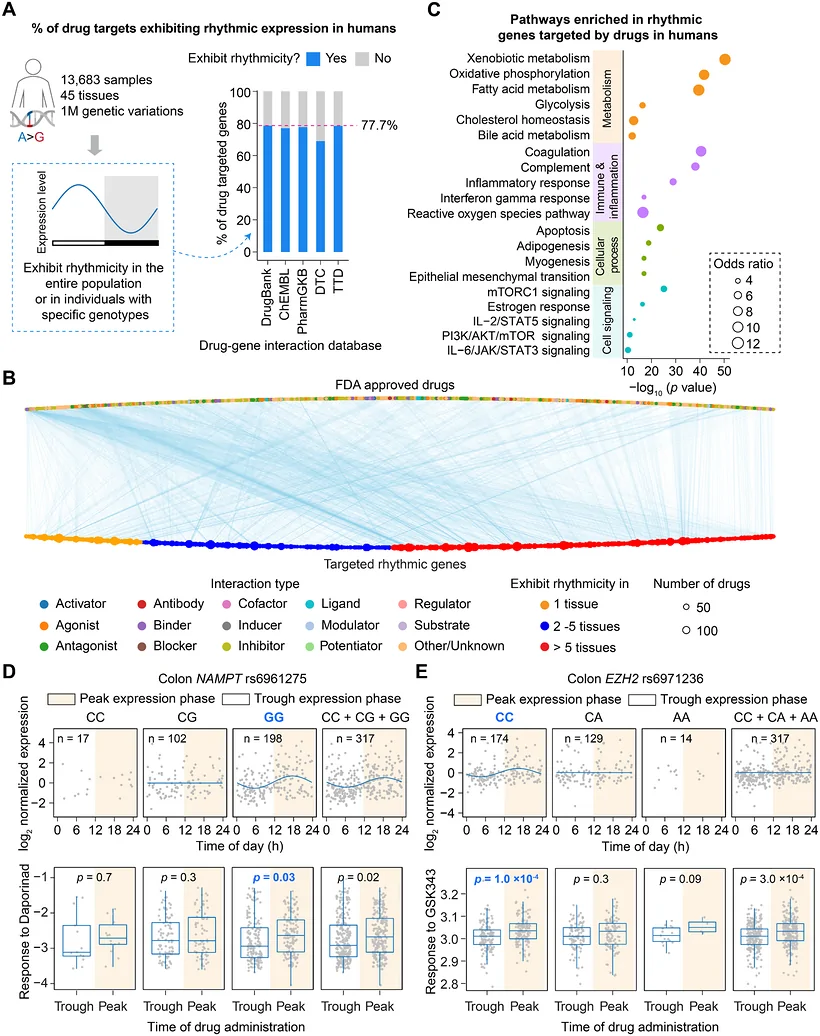
Expanding the landscape of therapeutic targets for precision circadian medicine by considering human genetic variations. (A) Percentage of drug targets with rhythmic expression in at least one human tissue. Rhythmic genes were defined based on genome‐wide time‐series transcriptomic analyses across 45 human tissues, including both canonical population‐level rhythmic genes and genotype‐dependent rhythmic genes identified through our previous rhythmic quantitative trait loci (rhyQTL) analysis. Drug–target annotations were obtained from five drug–gene interaction databases, including DrugBank, ChEMBL, PharmGKB, DTC, and TTD. (B) Global landscape of associations between drugs and their rhythmic target genes. Each line represents a drug–gene interaction, colored by interaction type, and target genes are annotated by the number of tissues in which rhythmic expression is observed. (C) Pathway enrichment analysis of rhythmic genes targeted by FDA‐approved drugs from the DrugBank database. Pathways are grouped into four functional categories, including metabolism, immune response, cancer‐related signaling, and cellular processes. The dot size represents the odds ratio of enrichment for rhythmic drug target genes in each pathway relative to the background gene set. (D, E) Genotype‐dependent variation in predicted drug response for Daporinad (D) and GSK343 (E). Upper panels show rhythmic expression patterns stratified by genotype groups, with trough phases highlighted by yellow shading. Peak and trough windows were defined as time intervals spanning ±6 h around the respective peak and trough phases. Lower panels show the comparison of predicted drug responses between peak and trough dosing windows. Genotype groups with fewer than 50 samples were excluded from rhythmicity analysis to avoid unreliable estimates due to small sample size.

Considering the impact of genetic variation on rhythmic gene expression, we recently identified a median of four times more rhythmic genes across 45 tissues than previously recognized in past population analyses [12]. This expanded set of human rhythmic genes provides new opportunities to identify additional druggable targets with rhythmic expression in individuals with specific genetic backgrounds. Here, we collected drug‐gene interactions from Drugbank [13], Chembl [14], PharmGKB [15], and Therapeutic Target Database [16] and matched genes in these interactions to all rhythmic genes identified across 13 683 samples representing 45 human tissues. Compared with prior estimates indicating that 56% of FDA‐approved drug targets [11] are rhythmic and 13% of circadian genes are targeted by approved drugs [11, 17], our integration revealed substantially higher coverage, with about 80% of FDA‐approved drug targets being rhythmic and about 30% of circadian genes mapped to at least one drug (Figure 1A,B). Notably, rhythmic genes are significantly enriched among drug targets compared to randomly selected genes (Figure S1A), indicating that circadian regulation is more prevalent among pharmacologically targeted genes. Rhythmic genes targeted by FDA‐approved drugs are significantly enriched in pathways related to metabolic processes (e.g., fatty acid metabolism, glycolysis, bile acid metabolism), immune and inflammatory responses (e.g., interferon‐γ response, complement activation, ROS pathway), cellular state transitions (e.g., apoptosis, adipogenesis, epithelial mesenchymal transition), and canonical signaling pathways (e.g., PI3K/AKT/mTOR, JAK/STAT, mTORC1, and estrogen response) (Figure 1C). Collectively, these findings expand the catalog of rhythmically druggable targets and broaden the scope for temporal optimization of drug administration.

To assess how genotype‐dependent rhythmicity influences drug response, we incorporated transcriptome‐based predictions of drug sensitivity. We used drug‐sensitivity data, represented by IC50 values, that were previously predicted for GTEx samples using a machine‐learning model [18]. The IC50 was defined as the concentration of a drug required to inhibit cell growth by 50%, with lower values indicating greater sensitivity. These predictions were generated for various drugs across GTEx samples based on the transcriptome profiles (Figure S1B). For each drug–target gene pair, we compared predicted drug sensitivity between peak and trough expression windows. This analysis supports the notion that time of day–dependent drug responses are influenced by rhythmic gene expression. For example, nicotinamide phosphoribosyltransferase, encoded by *NAMPT*, is inhibited by the drug daporinad and shows genotype‐dependent rhythmic expression at SNP rs6961275. Only the GG genotype group displayed rhythmicity of *NAMPT* and corresponding time of day–specific differences in predicted drug response (Figure 1D). Similarly, the epigenetic regulator *EZH2* exhibited rhythmic expression in individuals carrying the CC genotype at SNP rs6971236, which was associated with differential predicted sensitivity to its inhibitor, GSK343 (Figure 1E). Together, these findings show that germline variation generates genotype‐specific temporal windows of drug sensitivity, highlighting the relevance of circadian regulation to precision pharmacology.

### 2.2 Pan‐Cancer Circadian Analysis Uncovers Genes With Disrupted Rhythmicity That Represent Potential Chronotherapy Targets

**FIGURE 2:**
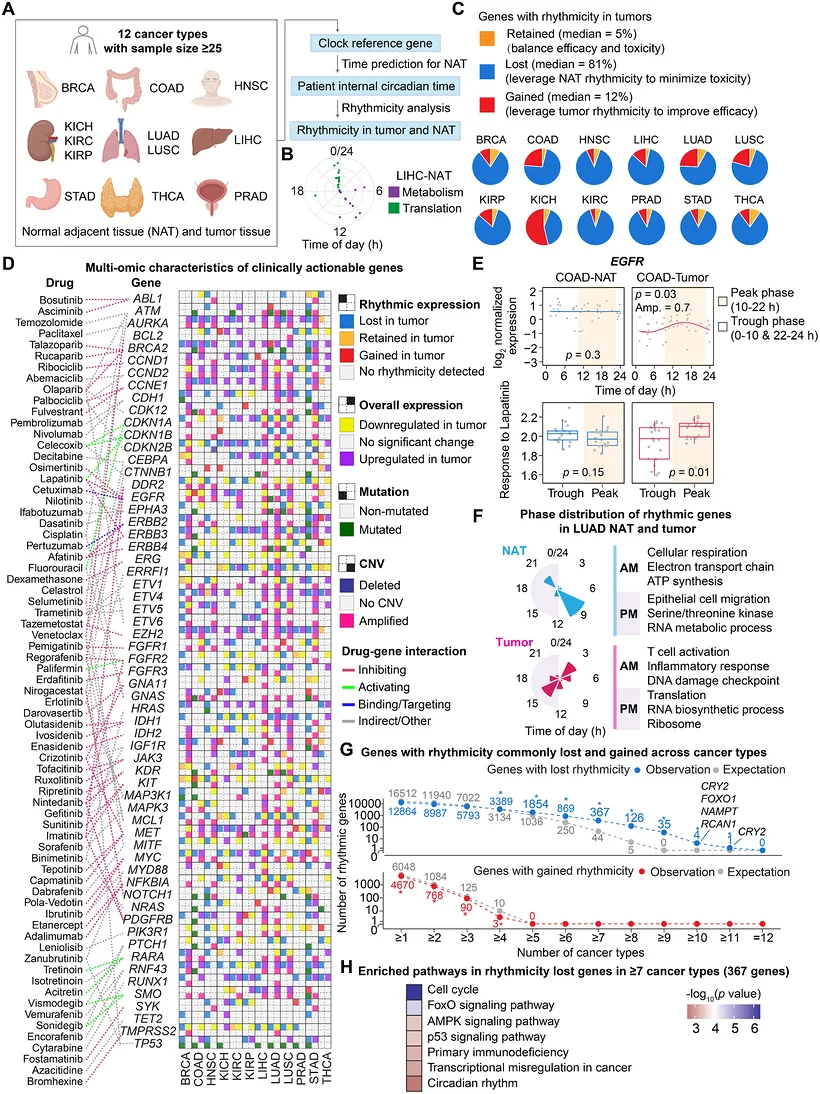
Pan‐cancer circadian analysis uncovers genes with disrupted rhythmicity that represent potential chronotherapy targets. (A) Schematic overview of circadian time inference and rhythmicity analysis across 12 TCGA cancer types with sample size ≥ 25. Internal circadian time for each sample was predicted using clock reference genes based on transcriptomic data from matched normal adjacent tissues (NAT), followed by rhythmicity analysis in both tumor and NAT samples. (B) Polar plots showing the phase distribution of rhythmic genes associated with metabolic (purple) and translational (green) pathways in liver adjacent normal tissue (LIHC‐NAT). Each point represents a rhythmic gene positioned according to its peak phase. Enrichment analysis indicates that metabolic pathways are enriched among genes peaking in the morning (0–12 h), whereas translational pathways are enriched among genes peaking in the afternoon (12–24 h). (C) Percentage of rhythmic genes across different cancer types, categorized into three groups based on changes in rhythmicity between tumor and matched NAT: retained (rhythmic in both), lost (rhythmic in NAT only), and gained (rhythmic in tumor only). (D) Multi‐omic characteristics of clinically actionable genes across cancer types. The drug–gene interaction network (left) and multi‐omic alteration profiles of clinically actionable genes across 12 cancer types (right) are shown. Each column represents a cancer type, and each row represents a gene. Color annotations indicate rhythmic expression status, gene expression changes relative to NAT, mutation status, and copy number variation across cancer types. (E) Rhythmic expression of EGFR in COAD‐NAT and COAD‐Tumor (top) and predicted responses to Lapatinib in patients with internal circadian phases at the trough vs. peak expression phases of EGFR (bottom). (F) Radial plots showing the distribution of the phases of rhythmic genes in LUAD, along with pathways enriched among genes peaking in the morning (0–12 h) and afternoon (12–24 h) phases. The phase distributions between tumor and NAT are significantly different in LUAD (*p* < 0.001, Watson's two‐sample test). (G) The number of genes that commonly lose or gain rhythmicity across different cancer types. The expected number of rhythmic genes (shown in grey) was determined by randomly sampling an equal number of expressed genes within each cancer type 1000 times while maintaining the empirical expression distribution and detection power. The expected values represent the median of the null distribution, and *p* values were computed by comparing the observed counts to the permutation‐derived distribution. ^*^ *p* < 0.05. (H) Enriched pathways in genes with rhythmicity lost in at least seven cancer types.

To further explore the application of rhythmic genes in circadian medicine under pathological conditions, we first focused on human cancers. We systematically profiled circadian gene expression across tumors and matched normal adjacent tissues (NATs) to define cancer‐associated alterations in rhythmicity. We analyzed 12 cancer types from The Cancer Genome Atlas (TCGA) with sufficient RNA‐seq samples (≥ 25) from both tumors and NATs (Figure 2A and see Methods for details) and inferred the internal circadian phase of each patient based on the relative expression of reference clock genes in NATs, as done in previous studies [12, 19, 20] (Figure 2A and detail in Methods). To test that the inferred phase captures physiologically relevant rhythmicity, we determined gene rhythmicity in liver normal adjacent tissue (LIHC‐NAT), a peripheral organ exhibiting robust circadian oscillations [21, 22]. We found that genes involved in metabolic pathways peaked during the daytime, whereas those associated with protein translation exhibited peak expression around midnight (Figure 2B and Table S1), consistent with prior observations in humans [23]. Moreover, the phases of these two pathways in humans displayed an approximately 12‐h shift relative to those observed in nocturnal mouse models [24].

After observing phase patterns consistent with known circadian biology in LIHC‐NAT, we next assessed rhythmic expression in other tumors and matched NATs (Figure 2A and see Methods for details). We found tumor tissues exhibited a widespread loss of rhythmic gene expression across all 12 cancer types, with a median of 81% of rhythmic genes losing their rhythmicity and only about 12% showing gain of rhythmicity compared to NATs (Figure 2C). For example, human liver cancer showed a marked reduction in the number of rhythmic genes (Figure S2A), which are involved in immune regulation, apoptosis, and lipid metabolism (Figure S2B). These findings suggest that tumorigenesis is commonly associated with circadian disruption, consistent with previous studies linking circadian clock dysfunction to cancer progression and metabolic deregulation [25, 26, 27]. Accordingly, retained rhythmicity may guide treatment timing to balance efficacy and toxicity, lost rhythmicity may allow leveraging preserved NAT temporal programs to minimize side effects, and gained rhythmicity may expose tumor‐specific temporal vulnerabilities for therapeutic exploitation (Figure 2C).

To explore potential translational applications, we focused on 135 clinically drug‐targetable genes associated with therapeutic decision‐making [26, 28]. Among these, 61 genes exhibited rhythmic expression in tumors or NATs across at least three types of cancer (Figure 2D). Extending beyond somatic mutations, copy‐number variation (CNV) and overall expression, we incorporated rhythmicity as a temporal layer, providing a multi‐omic view of how circadian reprogramming intersects clinically relevant targets to guide the temporal optimization of drug administration (Figure 2D). For example, *EGFR*, a key driver of MAPK and PI3K signaling and a target of multiple tyrosine kinase inhibitors, and *MET*, the receptor for HGF with several approved inhibitors in solid tumors, are both widely targeted in clinical cancer therapy [29, 30, 31]. *EGFR* in colon adenocarcinoma (COAD) and *MET* in thyroid carcinoma (THCA) gained rhythmicity in tumor tissues relative to NATs. Integrated drug‐response analyses indicated significant peak and trough differences in predicted drug response, with no corresponding effects in NATs (Figure 2E and Figure S2C).

Beyond rhythmicity, the circadian phase, defined by the timing of peak expression of rhythmic genes, is critical for coordinating metabolic and proliferative outcomes. Comparing phase distributions of rhythmic genes between tumors and NATs revealed substantial shifts across all cancer types (Figure 2F and Figure S2D, Watson's two‐sample test, *p* < 0.001). In lung adenocarcinoma NATs, rhythmic genes with morning phases are enriched for pathways related to cellular respiration, oxidative phosphorylation, and ATP synthesis, whereas those with afternoon phases are associated with epithelial migration and RNA‐metabolism programs (Figure 2F). In tumors, rhythmic genes with morning phases are enriched for T‐cell activation and inflammatory signaling pathways, while those with afternoon phases are associated with translational programs (Figure 2F). Notably, morning activation of the immune pathway aligns with previous findings of enhanced tumor immunity during the morning [25] and with clinical observations that earlier‐day immunotherapy improves efficacy and survival in lung cancer [32].

By defining circadian rhythms at the gene level, our approach enables the identification of genes that consistently lose or gain rhythmic expression across different cancer types. To determine whether these shared lost or gained rhythmic genes exceeded random expectation, we constructed an empirical null model by repeatedly sampling expression‐ and number‐matched genes. We observed a statistically significant excess of genes losing rhythmicity across multiple cancer types relative to this null model (Figure 2G and Figure S2D), suggesting a convergent disruption of circadian regulation during tumorigenesis. By contrast, genes that gained rhythmic expression were significantly fewer than expected, suggesting that newly acquired rhythmicity is largely cancer specific. To investigate the biological processes underlying this consistent loss of rhythmicity, we performed pathway enrichment analysis on 367 genes that lost rhythmicity in at least seven cancer types. These genes were significantly enriched in pathways related to cell cycle control, FoxO signaling, p53 response, and immune regulation (Figure 2H). Many of these pathways are known to be under circadian control and play critical roles in tumorigenesis [33, 34, 35]. We found four genes, *CRY2*, *FOXO1*, *NAMPT*, and *RCAN* *1*, consistently lost rhythmic expression in more than 10 cancer types, suggesting that they may play critical roles in tumorigenesis and could server as chronotherapeutic targets for these cancers (Figure 2G and Figure S2G–J). Indeed, CRY agonist, SHP1705, has been reported as an anti‐cancer reagent [36]. Together, these analyses add a temporal layer to clinical annotation and expand the targets for circadian medicine in cancer.

### 2.3 Cross‐Condition Analyses Reveal Conserved Disruption of Rhythmic Genes

**FIGURE 3:**
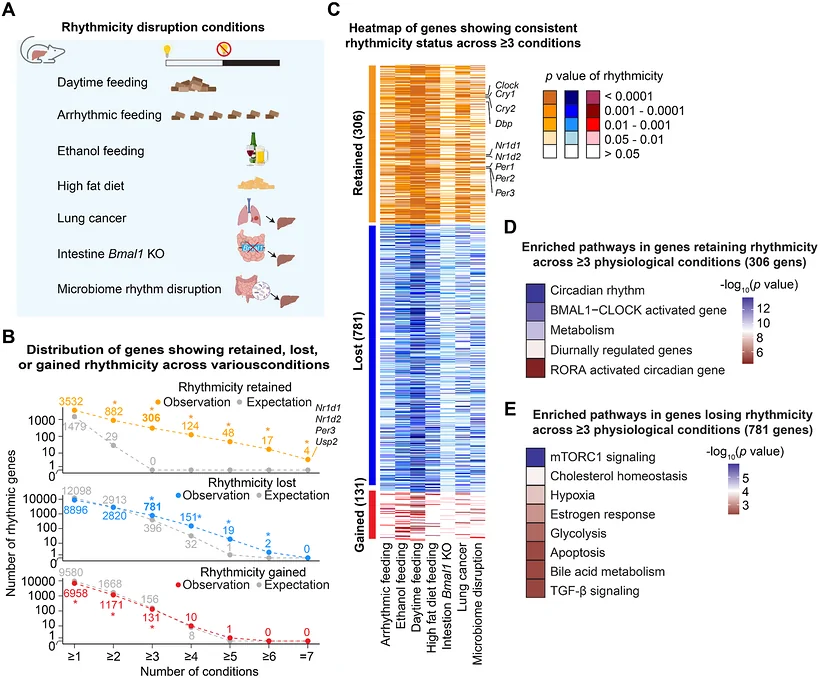
Circadian reprogramming across pathological conditions uncovers rhythmic genes implicated in physiological homeostasis. (A) Overview of seven pathological conditions in mice. (B) Number of rhythmic genes retained, lost, or gained under pathological conditions compared to control. The *p* value is determined by comparing observed gene counts to a null distribution from 1000 random samplings. ^*^ *p* < 0.05. (C) Heatmap of genes showing consistent rhythmicity status across conditions. Genes were classified as retained, lost, or gained based on comparisons between each control and pathological conditions, and genes exhibiting the same rhythmicity status in ≥ 3 conditions were selected for visualization. (D, E) Pathway enrichment analysis of genes with consistent rhythmicity changes across conditions. 306 genes classified as rhythmically retained in ≥ 3 conditions were used for enrichment analysis in (D), whereas 781 genes classified as rhythmically lost in ≥ 3 conditions were used in (E). All gene sets were derived from genes showing consistent rhythmicity changes across the seven pathological conditions.

To further determine whether shared lost rhythmic genes are critical for maintaining physiological homeostasis across different pathological conditions, we analyzed circadian transcriptomic data collected from genetically and environmentally well‐controlled preclinical mouse models. Consistent with findings in humans (Figure 1A), we found that approximately 80% of drug‐targeted genes in healthy mice exhibit rhythmic expression (Figure S3). We further examined how pathological conditions affect circadian dynamics by focusing on the mouse liver, the organ with the greatest number of rhythmic genes and a central metabolic hub. We analyzed seven distinct conditions involving nutritional challenges, disease models, genetic perturbations in other tissues, and microbiome disruption (Figure 3A).

Interestingly, a substantial number of rhythmic genes consistently lost their oscillatory expression across multiple pathological conditions in mouse livers, significantly exceeding random expectation, whereas shared gained rhythmic genes were markedly fewer than expected (Figure 3B,C). Notably, the number of rhythmic genes consistently retained across conditions was significantly higher than expected, suggesting a preserved rhythmic program under these pathological conditions, including core clock genes *CLOCK*, *PER2*, and *NR1D1* (Figure 3B,C). Based on the distribution (Figure 3B), we next examined genes showing consistent rhythmicity status across conditions. Genes displaying retained (306 genes), lost (781 genes), or gained (131 genes) rhythmicity in at least three disruption conditions were selected to represent robust rhythmic responses (Figure 3C). Functional enrichment analysis revealed that retained rhythmic genes were enriched in circadian regulation, metabolic pathways, and BMAL1–CLOCK targets (Figure 3D), whereas genes that lost rhythmicity across multiple conditions were enriched in stress‐related pathways, including mTORC1 signaling, estrogen response, hypoxia, and TGF‐β signaling (Figure 3E). Together, although circadian remodeling is observed across conditions, genes that lose rhythmicity are commonly shared across pathological states, whereas gains in rhythmicity remain largely condition specific. This pattern suggests that commonly lost rhythmic genes play important roles in maintaining physiological homeostasis.

### 2.4 Integrative Multi‐Omics Analyses Identified Upstream Circadian Regulators

**FIGURE 4:**
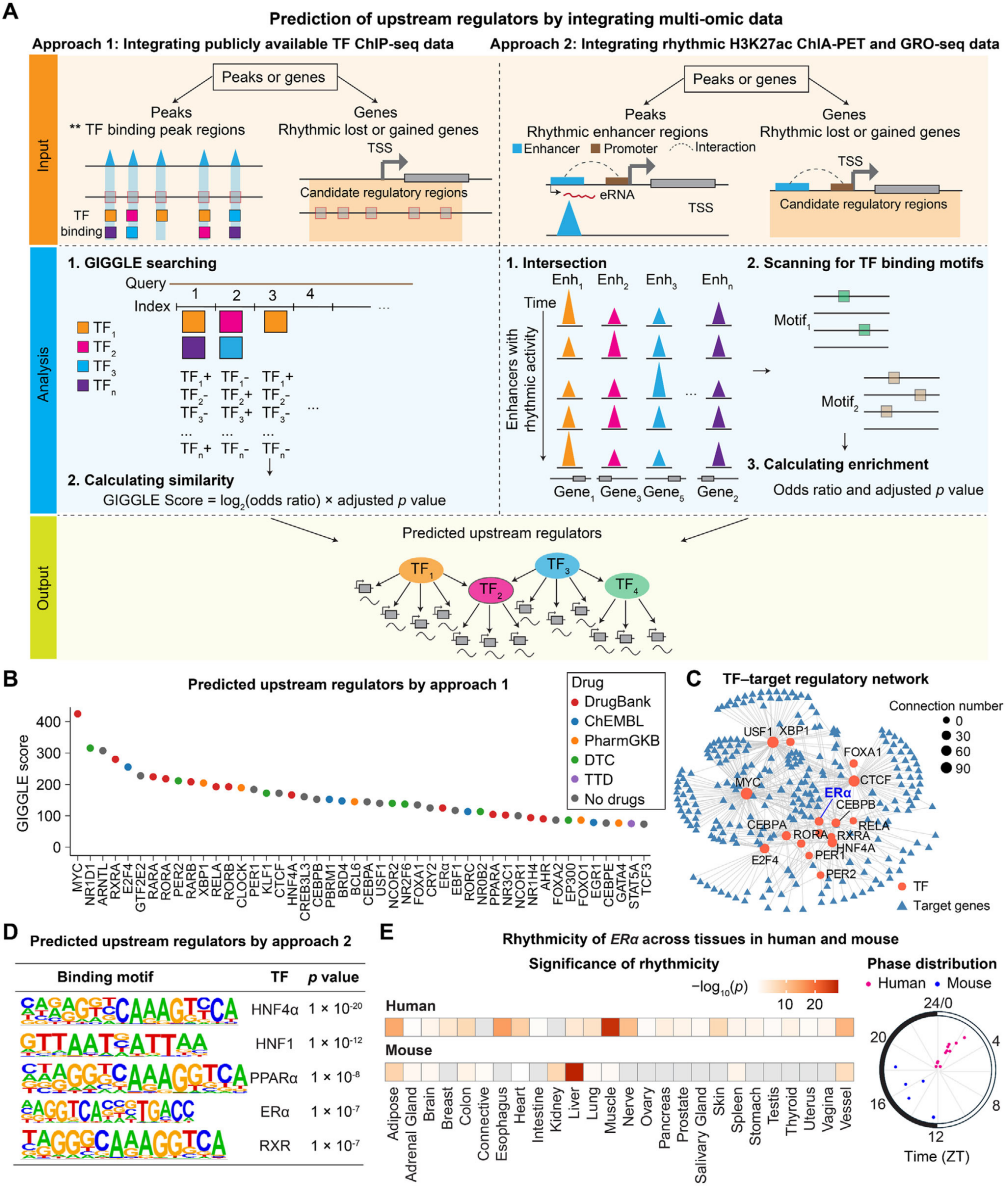
Integrative multi‐omics analysis identifies upstream clock regulators. (A) Schematic framework for identifying upstream regulators by integrating TF ChIP‐seq and rhythmic ChIA‐PET and GRO‐seq data. Two complementary approaches were used to infer candidate transcription factors regulating genes with altered rhythmicity. (B) Predicted upstream regulators based on TF ChIP‐seq data, ranked by GIGGLE score. (C) Transcription factor (TF)‐target gene regulatory network. Network visualization shows rhythmic transcription factors (orange circles) and their downstream target genes (blue triangles) that lose rhythmicity under more than three pathological conditions. Node size is proportional to the number of regulatory connections. (D) Predicted upstream regulators based on ChIA‐PET and GRO‐seq data, ranked by motif enrichment significance. (E) Rhythmicity of *ERα* across tissues in human and mouse. The left heatmap shows the rhythmicity significance (‐log_10_(*p*)) of the *ERα* gene. The circular plot on the right displays the phase distribution (i.e., peak expression time in circadian time, ZT) for tissues showing significant rhythmicity (*p* < 0.05) and peak‐to‐trough fold change > 1.5.

The widespread loss of rhythmic gene expression observed in human tumors and across various pathological conditions in mice raises the question of the underlying regulatory mechanisms and the specific transcriptional regulators responsible for these alterations. To address this, we developed a computational framework based on two complementary approaches to uncover rhythm‐associated regulatory factors (Figure 4A). We assessed whether specific transcription factors (TFs) exhibit enriched binding at *cis*‐regulatory elements (CREs) of genes that consistently lost rhythmicity across at least three pathological conditions. We leveraged high‐quality TF ChIP‐seq datasets from mouse liver and quantified the significance and enrichment of genomic overlaps using the GIGGLE score [37], a rank‐based metric that evaluates the co‐localization of TF binding sites with CREs of rhythmically disrupted genes (Figure 4A). We found that among the top‐ranked TFs were core clock genes, including NR1D1 (also known as REV‐ERBα) and ARNTL (also known as BMAL1) (Figure 4B). This finding confirms their central roles in driving circadian transcriptional programs [38, 39]. In addition to core clock genes, several other top‐ranked TFs, such as MYC and BRD4, also showed strong enrichment at CREs of common rhythmicity‐loss genes. These noncanonical clock regulatory TFs have been previously implicated in circadian regulation or metabolic reprogramming, highlighting their potential role in circadian reprogramming under pathological conditions [40, 41]. Notably, many of these TFs, such as RARA and RXRA, are known targets of FDA‐approved drugs [42, 43] (Figure 4B), highlighting their potential for therapeutic targeting and drug development. To further illustrate how these TFs coordinate downstream rhythmic disruption, we constructed a TF–target regulatory network, which revealed extensive connectivity between top‐ranked TFs and genes that commonly lost rhythmicity across pathophysiological conditions (Figure 4C).

Next, we mapped CREs with rhythmic transcription activities to genes that consistently lost rhythmic expression in pathological conditions by incorporating rhythmic chromatin interactions and enhancer activities (details in Methods). We scanned the CREs associated with these common lost rhythmic genes for enriched TF‐binding motifs using HOMER (Figure 4A). HNF4α, HNF1, PPARα, ERα, and RXR were identified as top regulatory TFs (Figure 4D). HNF4α, PPARα, and HNF1 have been previously implicated in circadian regulation and metabolic homeostasis under disease conditions [44, 45, 46]. Notably, RXR and ERα were enriched in both approaches. ERα is a hormone‐responsive transcription factor with a well‐defined role in energy balance and metabolic regulation. Estrogen replacement therapy (ERT), which acts through ERα, is widely used to alleviate menopausal and metabolic symptoms in postmenopausal women, including prevention of adiposity, hepatic steatosis, and metabolic inflexibility [47]. Importantly, estrogen response pathways, together with functionally coupled AKT‐related signaling programs, such as AMPK, FOXO, cell cycle, and mTORC1 signaling were consistently enriched across multiple layers of our analysis, including drug‐target genes with rhythmic expression in humans (Figure 1C), genes that lost rhythmicity under diverse pathological conditions in mouse liver (Figure 3E), and the predicted upstream regulators of common rhythmicity‐loss genes in human cancers (Figure S4A). Together, integrative analyses of transcriptomic and epigenomic data suggest a molecular framework in which ERα signaling, in coordination with AKT‐centered metabolic and cell‐cycle regulatory programs, contributes to the conserved disruption of rhythmic gene expression across pathological conditions (Figure S4B).

Moreover, *ERα* exhibited conserved rhythmic expression across multiple tissues in both humans and mice (Figure 4E). Aligned with the physically active phase (day vs. night), the phase of *ERα* in humans and mice is reversed, even though humans do not follow a strict 12‐h light:12‐h dark cycle (Figure 4E). This observed anti‐phase alignment underscores a conserved circadian regulation of *ERα* that is adapted to species‐specific behavioral rhythms. Consistent with this observation, estrogen signaling has been recognized as a key regulator of behavioral and peripheral circadian rhythms, primarily from studies using continuous estradiol replacement [48, 49, 50]. However, the functional significance of time‐of‐day–dependent estrogen signaling and its relationship to circadian regulation is still largely unknown. Therefore, we focus on the temporal dimension of estrogen responsiveness by administering estradiol at defined circadian phases to assess whether physiological responses depend on the timing of hormone exposure in ovariectomized (OVX) mice, a well‐established model of estrogen deficiency that recapitulates key features of the postmenopausal state.

### 2.5 Time‐of‐Day Differences in Metabolic Responses to Estrogen Administration

**FIGURE 5:**
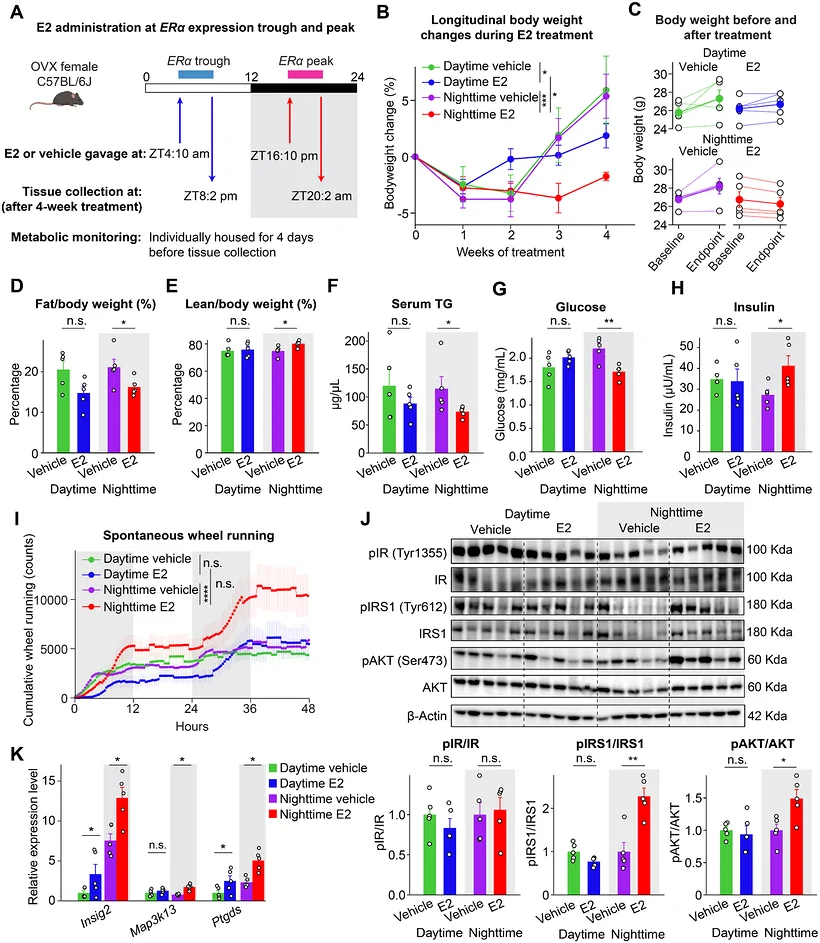
Nighttime E2 administration is associated with improved metabolic outcomes in OVX mice. (A) Experimental design. OVX mice received daily E2 or vehicle at ZT4 (ERα trough) or ZT16 (ERα peak) for 4 weeks. (B) Longitudinal body weight changes during E2 treatment, expressed as percentage change from baseline body weight. Data were analyzed using repeated‐measures two‐way ANOVA. (C) Individual paired body weight measurements at baseline and endpoint for each mouse. (D, E) E2 decreased fat/body weight ratio (main effect of treatment, *p* = 0.006, two‐way ANOVA), with no significant main effect of time or treatment × time interaction. No significant effects were observed for lean/body weight ratio (E). (F) Serum triglycerides showed a trend toward a main effect of treatment (*p* = 0.07), with no significant time effect or interaction. (G) Glucose levels showed a significant treatment × time interaction (*p* = 0.002), with no significant main effects of treatment or time. (H) Insulin levels showed a trend toward a treatment × time interaction (*p* = 0.08), with no significant main effects of treatment and time. (I) Cumulative spontaneous wheel‐running activity over 48 h. Data were analyzed using repeated‐measures two‐way ANOVA. (J) Hepatic insulin signaling analysis revealed a significant treatment × time interaction for IRS1 phosphorylation (*p* < 0.001), a significant main effect of treatment time (*p* = 0.04), and a trend toward a main effect of E2 treatment (*p* = 0.06). AKT phosphorylation showed a significant main effect of treatment (*p* = 0.04), a significant main effect of time (*p* < 0.001), and a significant treatment × time interaction (*p* = 0.02). IR phosphorylation showed no significant main effects or interaction. (K) E2 significantly upregulated insulin‐responsive genes (*Insig2*, *Map3k13*, *Ptgds*; all main effect of treatment, *p* ≤ 0.002). Significant main effects of time were observed for *Insig2* (*p* < 0.001) and *Ptgds* (*p* = 0.001), and a treatment × time interaction was detected for *Map3k13* (*p* = 0.03). For bar graph panels (D–H, J, and K), asterisks denote pairwise comparisons between indicated groups assessed by two‐tailed unpaired Wilcoxon rank‐sum tests. For panels (B, I), asterisks denote comparisons assessed by repeated‐measures two‐way ANOVA. Data are presented as mean ± SEM. *n* = 5 mice per group. *p < 0.05, ^**^ *p* < 0.01, ^***^ *p* < 0.001; n.s., not significant.

To determine whether the metabolic effects of estrogen depend on the timing of administration relative to ERα expression, OVX mice were treated daily with 17β‐estradiol (E2) or vehicle at either the trough (ZT4, daytime) or peak (ZT16, nighttime) of ERα expression for 4 weeks (Figure 5A). Body weight showed an initial decline during the first week of treatment across all groups, likely reflecting physiological adaptation to the daily gavage procedure. From week 2, group‐specific trajectories became apparent, with nighttime E2 treatment resulting in a more sustained reduction in body weight compared with daytime E2 administration (Figure 5B).

Body weight was comparable across groups both at the time of OVX surgery and at the start of E2 treatment (Figure 5C, Figure S5A,B and Table S2), indicating that the observed differences were not driven by baseline variability. Paired comparisons of baseline and endpoint measurements showed a larger reduction in body weight gain and fat/body weight ratio in the nighttime E2 group relative to daytime E2 (Figure 5D and Figure S5C). Two‐way ANOVA with treatment (E2 vs. vehicle) and time (daytime vs. nighttime) as independent variables revealed a significant main effect of E2 treatment on body weight change (*p* = 0.009) and fat/body weight ratio (*p* = 0.006), with no significant main effect of time or treatment × time interaction for either measure. Lean/body weight ratio showed no significant effect of treatment, time, or interaction (Figure 5E).

Nighttime E2 administration was associated with improved metabolic outcomes. Serum triglyceride levels showed a trend toward a main effect of E2 treatment by two‐way ANOVA (*p* = 0.07; Figure 5F), with lower levels in both E2‐treated groups compared with their respective vehicle controls. Nighttime E2‐treated mice showed significantly lower serum glucose compared with nighttime controls, whereas no significant difference was observed between daytime E2 and daytime vehicle groups. Two‐way ANOVA confirmed a significant treatment × time interaction for serum glucose (*p* = 0.002; Figure 5G), indicating that the glucose‐lowering effect of E2 is specific to nighttime administration. Serum insulin showed a trend toward a treatment × time interaction (*p* = 0.08; Figure 5H), with nighttime E2‐treated mice showing higher circulating insulin alongside lower glucose, consistent with improved insulin sensitivity rather than increased insulin secretion.   Consistent with the improvement in metabolic outcomes, nighttime E2 treatment increased spontaneous wheel‐running activity compared with nighttime controls (Figure 5I). Food intake did not differ significantly across groups, with no significant main effect of treatment, time, or interaction (Figure S5D), indicating that the observed metabolic differences are unlikely to be attributable to altered caloric intake.

To explore the molecular basis of these metabolic effects, we examined hepatic insulin signaling (Figure 5J and Figure S6). IR phosphorylation showed no significant main effect of treatment, suggesting that E2 does not substantially alter receptor‐level activation. In contrast, IRS1 phosphorylation showed a significant treatment × time interaction (*p* < 0.001), a significant main effect of treatment time (*p* = 0.04), and a trend toward a main effect of E2 treatment (*p* = 0.06). AKT phosphorylation showed a significant main effect of treatment (*p* = 0.04), a significant main effect of time (*p* < 0.001), and a significant treatment × time interaction (*p* = 0.02), indicating that the effect of E2 on AKT activation depends on the timing of administration. Together, these results are consistent with E2 modulating insulin signaling at a post‐receptor level. These signaling data suggest that nighttime E2 enhances hepatic insulin sensitivity through the IRS1–AKT signaling axis, providing a molecular correlate for the observed lower glucose levels.

Hepatic expression of ERα target genes further supported regulation by both E2 treatment and time of administration (Figure 5K). *Insig2* showed significant main effects of treatment (*p* = 0.001) and time (*p* = 5.32 × 10^−^^7^), with the highest expression in the nighttime E2 group. *Ptgds* showed significant main effects of treatment (*p* = 0.0004) and time (*p* = 0.0009). *Map3k13* showed a significant main effect of treatment (*p* = 0.0005) and a significant treatment × time interaction (*p* = 0.03).

Together, these findings suggest that metabolic and molecular responses to E2 are influenced by the timing of administration, with nighttime delivery associated with greater reductions in body weight, lower circulating glucose levels, and enhanced hepatic insulin signaling. Notably, time‐of‐day–dependent differences were more readily detected in longitudinal trajectories and molecular readouts than in endpoint measures of body composition, highlighting the importance of temporal dynamics in metabolic regulation.

### 2.6 An AI‐Powered Circadian Multi‐Omics Platform Supporting Integrative Analysis and Target Discovery in Circadian Medicine

**FIGURE 6:**
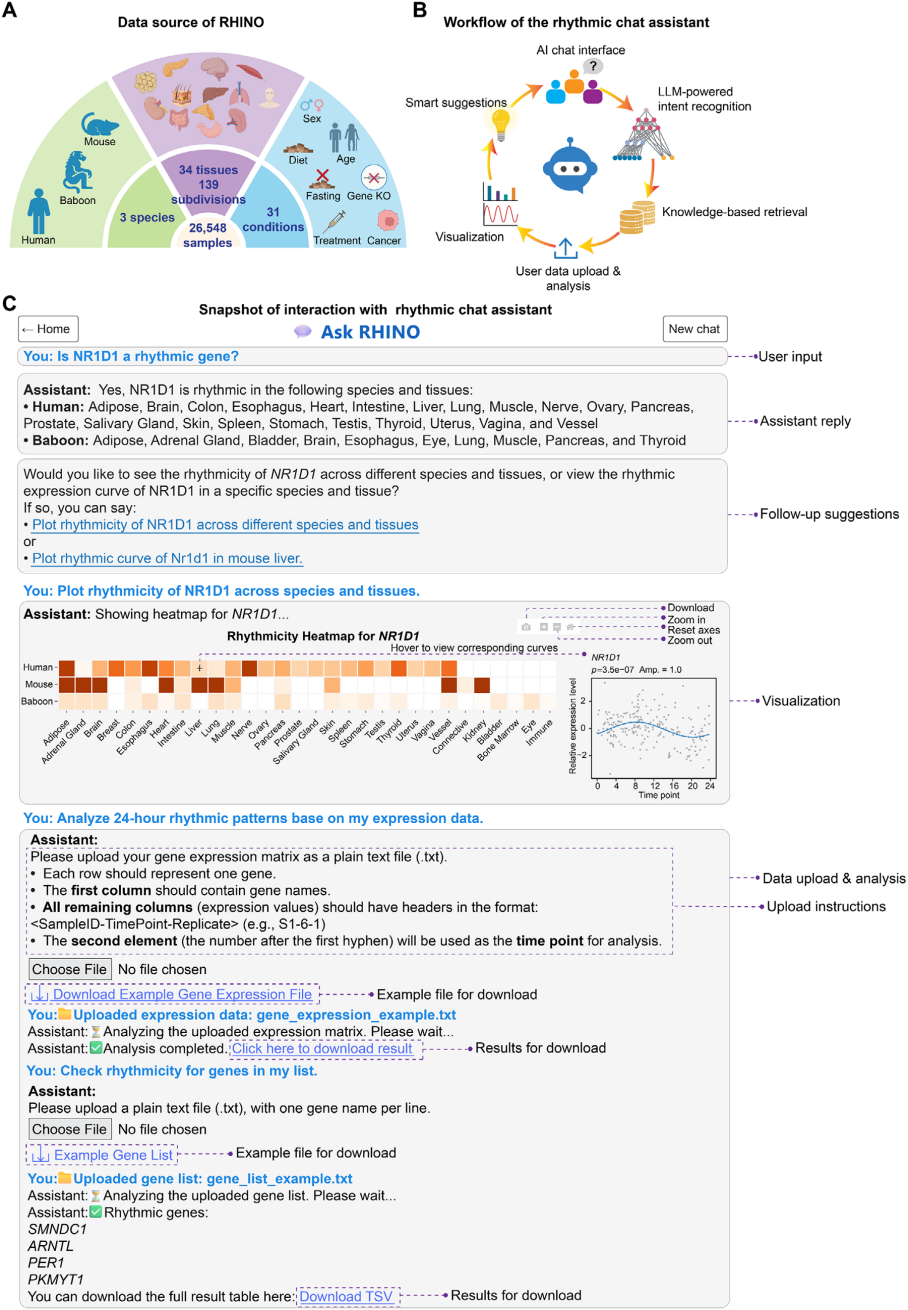
Overview and interaction of the RHINO platform. (A) Data sources integrated into RHINO, including multi‐species transcriptomic data across tissues, physiological conditions, and perturbations. (B) Workflow of the rhythmic chat assistant. The system integrates LLM‐based intent recognition with knowledge‐based retrieval and rhythmicity analysis to process user queries, generate responses, and produce visualizations. (C) Example interaction with the rhythmic chat assistant. The interface illustrates user input, assistant responses, follow‐up suggestions, visualization of rhythmic patterns, and data upload and analysis functions.

Despite growing recognition that circadian rhythms influence drug efficacy and toxicity, there remains a lack of integrated and accessible platforms for systematically exploring druggable targets in circadian medicine. As a result, our ability to fully leverage temporal biological insights for precision therapeutics and the development of chronotherapy approaches remains limited. To fill this gap, we developed RHINO (https://hanlaboratory.com/RHINO), a comprehensive web‐based platform designed to support both hypothesis‐driven and discovery‐based investigations of circadian regulation and its therapeutic implications (Figure S7A). RHINO is built upon a large‐scale multi‐omics compendium encompassing ten regulatory layers derived from 26 548 samples across 34 tissues and 139 anatomical subdivisions, spanning 31 genetic, pathological, and physiological conditions in humans, baboons, and mice (Figure 6A). This compendium integrates both the datasets analyzed in this study and additional publicly available multi‐omics resources that were curated and harmonized into a unified framework. A total of 18 data modalities capture diverse regulatory dimensions, including genetic variation, epigenomic and chromatin modifications, transcriptional and post‐transcriptional regulation, translation, protein abundance, and post‐translational modifications (Figure S7B). In addition to existing datasets, we also mapped rhythmic alternative polyadenylation (APA) events across diverse tissues (Figure S7C–E) to support the regulatory function of APA in controlling circadian processes [51, 52], highlighting RHINO's utility for exploring additional regulatory layers.

RHINO enables flexible visualization, diurnal oscillation detection, and comparative exploration across species and conditions, providing a unified platform for advancing multi‐layered circadian biology. Through an intuitive interface, users can explore gene‐specific rhythmicity across species, tissues, and regulatory levels (Figure S8A,B), examine genetic variants associated with circadian expression via the integrated genetic variation module (Figure S8C), and conduct customized analyses by uploading gene lists or time‐series expression matrices (Figure S9). Building on these capabilities, we further enhance the user experience through an intelligent conversational interface.

To meet the demand for intuitive, intelligent, and accessible biological data exploration, RHINO integrates an AI chat module designed to bridge large‐scale rhythmic datasets with natural language interaction (Figure S7A). This module was developed in response to recent advances in AI and user expectations for more conversational and flexible interfaces in scientific analysis. At its core, the AI chat is powered by a large language model (LLM)‐based conversational assistant [53], which combines natural language understanding, real‐time computation, and biological knowledge retrieval to support both precise and exploratory circadian queries (Figure 6B). A typical interaction begins with a free‐form user query (e.g., “Is *NR1D1* rhythmic?”), to which the assistant replies with a combination of concise explanations, rhythmic heatmaps, and fitted circadian expression curves across species and tissues (Figure 6C). In addition, users can upload their own gene expression matrices or gene lists, which are automatically parsed and analyzed on the backend. Results are returned in real time, including rhythmicity significance, amplitudes, phase estimates, and downloadable visualizations (Figure 6C). Overall, RHINO empowers both domain experts and non‐specialists to investigate circadian patterns with minimal computational effort. It offers a scalable, intelligent, and user‐friendly platform for high‐throughput circadian data mining, hypothesis generation, and translational exploration.

## 3 Discussion

Circadian medicine offers a promising and low‐burden approach for optimizing therapeutic interventions by aligning drug administration with the body's intrinsic biological rhythms. To expand the druggable targets for circadian medicine, we performed integrative multi‐omics analyses. Considering human genetic variations and rhythmic gene–drug pairs, we increased the proportion of FDA‐approved drugs suitable for time‐optimized therapy from previously reported 56% [11] to 80%. Beyond expanding target coverage, our analyses introduce rhythmicity as a temporal layer for clinically actionable genes. While prior studies have established circadian disruption in cancer [26, 54, 55], extending circadian analyses to clinically relevant gene–drug relationships facilitates translation toward time‐optimized therapeutic strategies.

By comparing rhythmic genes across different cancer types and pathophysiological conditions, ranging from nutritional challenges to genetic perturbations and microbiome disruption, we identified genes consistently losing rhythmic expression. The rhythmicity of these genes may be essential for maintaining physiological homeostasis, and restoring their rhythmic regulation could represent a novel therapeutic strategy. By integrating epigenomic changes, transcription factor binding motifs, and rhythmic transcriptomic data, we identified putative transcriptional regulators of these common rhythmicity‐loss genes and experimentally validated the time‐optimized metabolic benefits of ERα activation.

Clinical studies, including those involving statin therapy, immunotherapy, and time‐restricted eating, have demonstrated the beneficial effects of optimized timing of administration [6, 32, 56]. However, the results are not always consistent, whereas such inconsistencies are less frequently observed in preclinical animal models. In addition to individual lifestyle differences, our study suggests that genetic variations should also be considered, as exemplified by Daporinad and GSK343 in Figure 1D,E. Although precision medicine, tailoring treatment based on an individual's genetic background, has been well established, precision circadian medicine is still in its early stages and requires further investigation to improve the consistency of time‐optimized medical interventions in humans, as mentioned above.

Since the molecular cloning of the *Period* gene in Drosophila over 40 years ago, it has been recognized that core clock components form a transcription–translation feedback loop that regulates rhythmic gene expression and biological processes [1, 57]. These fundamental mechanistic insights led the studies on the role of core clock genes in human cancers and other diseases in previous studies [26, 54, 55]. However, recent transcriptome‐wide studies have revealed that hundreds of genes can retain rhythmic expression even in animal models lacking these core clock components [39, 58, 59]. Moreover, under various pathological conditions, thousands of genes lose or gain rhythmic expression while the core clock genes remain robustly rhythmic [45, 60]. These emerging findings suggest the existence of noncanonical clock regulators that modulate circadian processes. By comparing rhythmic genes across 12 different types of cancer and multiple pathophysiological conditions, we found multiple genes that consistently lost their rhythmic expression, suggesting their critical role in maintaining physiological homeostasis. By integrating epigenetic, cistromic, and transcriptomic analyses, we identified noncanonical clock regulators, including HNF4α, PPARα, and ERα.

Diurnal rhythms of circulating estrogen have been reported for decades, and the timing of estrogen induction in the day could influence ovulation [61, 62]. Moreover, while ERα has been shown to bind the promoter of Per2 and regulate its expression, these studies have primarily focused on the effects of overall estrogen levels or genetic knockout of ERα in disease etiology or hormone replacement therapy [63]. The functional significance of diurnal rhythms of circulating estrogen, particularly in metabolic regulation, still lacks direct experimental evidence. Our findings suggest that E2 treatment administered at night results in improved metabolic outcomes compared with daytime treatment. Increased IRS1 and AKT phosphorylation in nighttime E2‐treated mice, in the absence of a significant change in IR phosphorylation, is consistent with enhanced downstream insulin signal propagation. This pattern is consistent with previous studies showing that estrogen improves hepatic insulin sensitivity through ERα‐dependent activation of the IRS1–PI3K–AKT pathway [64]. Together, these observations support a model in which circadian variation in estrogen signaling contributes to time‐of‐day–dependent differences in metabolic outcomes. Since estrogen treatment is not only used for metabolic disorders, but also for cancers and women's health, the time‐dependent beneficial effects of estrogen treatment on these diseases still need to be determined in future studies. In this study, we used the OVX mouse model as a clinically relevant model of estrogen deficiency to explore time‐of‐day–dependent estrogen signaling. The OVX model was selected because it provides a physiologically integrated system in which circadian, hormonal, and metabolic signals interact in vivo, aspects that cannot be fully recapitulated in cell‐based models. Tissue‐specific perturbation models, such as liver‐specific *ERα* knockdown, will be important to directly establish the causal contribution of ERα signaling in specific metabolic tissues to the observed time‐of‐day–dependent effects. Future studies using disease‐specific models and incorporating mechanistic dissection will be important to further evaluate the therapeutic implications of circadian‐timed estrogen interventions.

To facilitate these analyses and support broader exploration of circadian regulation, we developed the RHINO platform, which integrates large‐scale circadian datasets across tissues, biological conditions, and multiple molecular layers. Compared with existing circadian resources (Table 1), RHINO integrates a larger and more diverse collection of circadian datasets and enables integrative analyses across regulatory layers and pathophysiological contexts. In addition, we implemented an AI‐assisted chatbot that allows users to explore circadian regulation and potential therapeutic targets related to diseases, genes, and regulatory molecules of interest. Together, these features provide a comprehensive framework for investigating circadian regulation and translating temporal biology into more precise and time‐informed therapeutic strategies.

**TABLE 1:** Summary of existing circadian research resources.

Database	Species	Total samples	Data type	Biological condition	Multi‐layer integration	Release / updates	PMID
RHINO	3 (mouse, baboon, human)	26 548	18 types of ‐omics with pharmacologic annotation^a^	31	Yes (with AI‐supported)	2025	This study
CircaKB	15	5577	9 types of ‐omics^b^	23	No	2024	39329269
RhythmicDB	19	∼1400	1 type (transcriptomic)	Physiological baseline	No	2022	35774515
CirGRDB	2 (mouse, human)	4936	9 types of ‐omics^c^	4 (physiological baseline, sleep disorder, aging, tumor)	Yes	2018	29059379
CGDB	148	Literature annotations, no time‐series data	1 type (transcriptomic)^d^	Physiological baseline	No	2016	27789706
CircadiOmics	16	6575	4 types of ‐omics^||^	20	No	2012, last updated 2022	22847108 35657089
CircaDB	2 (mouse, human)	∼5400	1 type (transcriptomic)	2 (Physiological baseline, genetic perturbations)	No	2012, last updated 2018	23180795

^a^ Genetic variation, epigenomic, transcriptomic, APA, translational, proteomic, post‐translational modification (PTM), and metabolomic data.

^b^ Bulk and single‐cell transcriptomics, nascent transcription, chromatin accessibility, translational profiling, and small RNA data.

^c^ Epigenomic and epitranscriptomic modifications (RNA editing and RNA methylation).

^d^ No circadian oscillatory traces or temporal dynamics are reported. || Transcriptomic, proteomic, acetylomic, and metabolomic data.

Several limitations of the present study should be noted. First, circulating E2 concentrations were not measured at specific timepoints following oral administration. Orally administered E2 in rodents exhibits transient peaks followed by a rapid decline within hours of administration [65]. Consistent with this pharmacokinetic profile, mice gavaged at ZT4 would be expected to have substantially lower circulating E2 by ZT20 (∼16 h post‐dose), whereas mice gavaged at ZT16 would remain within the post‐dosing exposure window at ZT20 (∼4 h post‐dose). Consequently, we cannot exclude pharmacokinetic differences as a contributing factor to the observed effects. Future studies incorporating time‐resolved circulating E2 measurements at defined timepoints, including ZT10 and ZT20, will be important to dissociate pharmacokinetic from pharmacodynamic contributions and clarify the mechanisms underlying time‐of‐day–dependent estrogenic effects. Second, while our findings demonstrate time‐of‐day–dependent differences in metabolic responses to E2, the underlying mechanisms were not fully disentangled in the current study. In particular, the relative contributions of circadian regulation, feeding behavior, and tissue‐specific signaling remain to be systematically characterized. Additionally, the absence of an intact reference group limits direct comparison between exogenous and endogenous estrogen signaling patterns, and future studies incorporating intact animals would provide important context for interpreting the OVX model findings. Reverse‐cycle feeding studies in OVX mice would provide important mechanistic insight into the relative contributions of feeding time vs. ERα expression rhythms to the observed metabolic outcomes and represent a valuable future direction. Third, molecular readouts such as AKT phosphorylation reflect signaling activity at a single timepoint rather than direct assessment of whole‐body metabolic states, and future studies incorporating comprehensive metabolic phenotyping will be important to further refine mechanistic interpretation. Finally, most functional validations were performed in mouse models, and the translational relevance of these findings to human physiology will require further investigation through clinical studies and well‐controlled chronopharmacological trials.

## 4 Materials and Methods

### 4.1 Data Sources

To comprehensively investigate circadian gene regulation across multiple molecular layers, we curated datasets from publicly available resources, including the Gene Expression Omnibus (GEO), The Cancer Genome Atlas (TCGA), the Genotype‐Tissue Expression (GTEx) project, CistromeDB, ENCODE, and peer‐reviewed literature. These datasets encompass diverse omics layers, including genotype, epigenome, transcriptome, translatome, post‐translational modifications (PTMs), and metabolome, thereby enabling integrative multi‐omics analysis. Genotype data for GTEx individuals were obtained via dbGaP (accession number: phs000424.v8.p2). A complete list of data sources and accession identifiers is provided on the “Datasets” page of our website (https://hanlaboratory.com/RHINO/Dataset.html).

To construct a cross‐species rhythmic multi‐omics dataset suitable for integration with drug annotations, we focused on mammalian species commonly used in pharmacological and circadian research. Accordingly, we systematically queried NCBI GEO for datasets involving *Homo sapiens*, *Mus musculus*, and *Papio anubis*. The search targeted studies annotated with circadian or diurnal keywords (e.g., “circadian,” “rhythm,” “clock,” “diurnal”) and covered a wide range of omics technologies, including transcriptomics (e.g., RNA‐seq, Poly(A)‐seq, Total RNA‐seq, microarray), translatomics (e.g., Ribo‐seq), epigenomics (e.g., ChIP‐seq, ChIA‐PET, Hi‐C, and GRO‐seq), proteomics (e.g., proteomics, PTM profiling including phosphorylation, ubiquitination, succinylation, and glycosylation), metabolomics, and lipidomics. We then manually curated these entries to retain only those with clearly defined time‐series designs or circadian perturbations (e.g., light/dark cycles, genetic modifications, and diets) for downstream integration. A total of 727 datasets were retrieved, encompassing 26 548 samples across 34 tissues and 139 anatomical subdivisions from humans, baboons, and mice under 31 genetic, pathological, and physiological conditions. We also included ChIP‐seq–based TF binding profiles and histone modification data from CistromeDB and ENCODE. Specifically, we focused on BED files reporting peak calls generated under wild‐type and normal chow conditions to reflect baseline regulatory activity. To ensure data quality, only datasets meeting all predefined quality control (QC) criteria in CistromeDB were retained [66]. After filtering, a total of 114 high‐quality TF ChIP‐seq datasets, as well as narrow peaks of histone modifications, including H3K27ac, H3K27me3, H3K36me3, H3K4me1, H3K4me2, H3K4me3, H3K79me2, H3K9ac, and H3K9me3, were incorporated across different tissues.

### 4.2 Data Processing

To evaluate potential regulatory mechanisms underlying rhythmic gene expression, we analyzed epigenomic features, including TF binding sites and histone modification marks, using ChIP‐seq data. Specifically, we assessed the genomic overlap between ChIP‐seq peaks and regulatory regions surrounding annotated transcripts. For each gene, a ±50 kilobase (kb) window centered on the transcription start site (TSS) was defined to capture both proximal promoter regions and distal *cis*‐regulatory elements, such as enhancers. Overlaps were used to infer potential regulatory relationships between epigenomic features and gene rhythmicity.

For transcriptomic analyses, raw RNA‐seq data downloaded from GEO in Sequence Read Archive (SRA) format were converted to FASTQ files using the SRA Toolkit. Low‐quality bases and adaptor sequences were removed using *fastp* (v0.20.0) with default parameters [67]. The retained high‐quality reads were then aligned to the appropriate reference genomes using the STAR aligner (v2.7.3a) [68]: the human genome (GRCh38, GCA_000001405.15), the mouse genome (GRCm38, GCA_000001635.8), or the baboon genome (Panu_3.0, GCA_000264685.2). BAM files were used for downstream detection of alternative polyadenylation (APA) events. Following alignment, transcript assembly and quantification were performed using *StringTie* (v2.2.1) [69], producing transcript‐level abundance estimates expressed as transcripts per million (TPM). These TPM values served as input for downstream rhythmicity detection analysis.

### 4.3 Detection of Alternative Polyadenylation (APA) Events

To expand the functional relevance of circadian regulation across molecular layers, we systematically identified rhythmic APA events. Circadian APA has recently been linked to sleep regulation and APA‐associated brain disorders [51, 52], underscoring its biological importance. To investigate rhythmic regulation at the level of 3′ untranslated regions (3′UTRs), we applied a standardized analysis pipeline to publicly available RNA‐seq datasets to identify APA events. Briefly, BAM files were first converted to WIG format using bedtools genomecov (v2.31.1), enabling base‐resolution read coverage along gene bodies. APA events were then inferred using DaPars (v2.1), which implements a linear regression model to identify dynamic changes in 3′UTR length and calculate the Percentage of Distal polyA site Usage Index (PDUI) for each transcript in every sample [70]. The PDUI was used to quantify the relative usage of distal vs. proximal polyadenylation sites. To evaluate potential circadian regulation of APA, PDUI values were standardized across time points using z‐score transformation, and the resulting normalized values were used for subsequent APA rhythmicity analysis.

### 4.4 Assessment of Rhythmicity

For mouse, baboon, and human datasets with well‐defined time‐series profiles (i.e., known sampling time points) across transcriptomic and other omics layers, we assessed molecular rhythmicity using the *MetaCycle* R package (v1.2.0) [71]. This package integrates three complementary algorithms, including ARSER, JTK_CYCLE, and Lomb‐Scargle, to identify genes with statistically significant 24‐h oscillations, providing robust estimation of period, phase, amplitude, and rhythmicity *p* values. *MetaCycle* outputs algorithm‐specific results, including raw *p* values, Benjamini‐Hochberg (BH) adjusted *q* values (FDR), phase (peak expression time point), and amplitude for each method. In addition, the meta2d function aggregates results from all available algorithms and reports consensus values for rhythmicity *p* value (meta2d_pvalue), phase (meta2d_phase), and amplitude (meta2d_AMP). We used these as parameters in our analysis for rhythmicity evaluation if meta2d results were available, as they represent a consensus across different detection methods. For datasets or genes where meta2d results were not available (e.g., due to partial failure of component methods), we defaulted to using the outputs from the JTK_CYCLE.

For human transcriptomic data retrieved from TCGA and GTEx, direct time‐of‐day sampling was not available. To estimate gene rhythmicity, we first inferred each individual's internal circadian phase. Prior to individual phase estimation, potential batch effects and confounding factors were addressed by regressing out known covariates using a linear model. For GTEx data, we included five genotype principal components (PCs), sequencing platform, library preparation protocol, ischemic time, age, sex, and type of death as covariates, following the approach described previously [12]. For TCGA data, the covariates included sequencing platform, age, and sex. Internal circadian phase was then predicted using the CHIRAL algorithm [19], which estimates biological time based on the normalized expression of 12 clock reference genes: *ARNTL*, *NPAS2*, *NR1D1*, *NR1D2*, *CIART*, *PER1*, *PER2*, *PER3*, *CRY1*, *CRY2*, *DBP*, and *TEF*.

Due to the non‐uniform distribution of sampling times across individuals, we modeled gene expression as a function of rhythmic phase using a cosinor regression model, which fits a sinusoidal curve with a fixed 24‐h period. This approach enables estimation of amplitude and phase for each gene while testing for significant rhythmicity under irregular time distributions. Specifically, gene expression values  *E_i_* of individual *i* were modeled as:

$$E_{i}=\mu +a\cos\left(\frac{2\pi t_{i}}{T}\right)+b\sin\left(\frac{2\pi t_{i}}{T}\right)+\varepsilon$$

where μ is the baseline expression level, *t_i_* is the predicted time (in hours) of individual *i*, *a* and *b* are coefficients used to compute the amplitude and phase of each gene, T is the period which is 24 h in this study, and ε represents residual noise. The peak‐to‐trough amplitude is calculated as $2\sqrt{a^{2}+b^{2}},$ and the phase was determined as the arctangent of the sine‐to‐cosine ratio $\frac{T}{2\pi }atan2\left(b_{xy},a_{xy}\right)\;mod\;T$ Rhythmicity was further assessed using a likelihood ratio test to compare the periodic model to a flat model (a = b = 0), assuming constant expression. The significance of the periodic component was determined by calculating the *p* value using the lmtest::lrtest function in *R*.

Genes were considered rhythmic if they exhibited significant 24‐h oscillations, defined by *p* value < 0.05 and amplitude > 1.5. For datasets with available sampling time information, including mouse and baboon datasets, rhythmicity was assessed using MetaCycle‐based analyses, and *p* values were derived from the meta2d function. For human datasets without direct sampling times, such as TCGA and GTEx, rhythmicity was assessed using a cosinor regression model with the same significance criteria.

### 4.5 Identification of Drugs Targeting Rhythmic Genes

To identify drugs with potential for time‐of‐day–optimized administration, we analyzed the pharmacological relevance of rhythmically expressed genes. We integrated drug annotation data from five major public databases: DrugBank, ChEMBL, PharmGKB, Drug Target Commons (DTC), and the Therapeutic Target Database (TTD). These resources provide complementary information on approved drugs, bioactive compounds, genetic associations, interaction strengths, and disease relevance. From DrugBank, we retrieved drug structures, therapeutic categories, and annotated targets, retaining entries explicitly labeled as “approved” for downstream analysis. These curated drug–target relationships were then cross‐referenced with rhythmically expressed genes identified in each species and tissues. We classified a drug to be time‐optimizable if its target gene shows rhythmic expression in at least one tissue.

### 4.6 Time‐of‐Day‐Dependent Drug Sensitivity Analysis

To evaluate the potential impact of rhythmic gene expression on drug sensitivity, we integrated transcriptome‐based drug sensitivity predictions from a previous study [18]. In the study, machine learning models were trained using gene expression and drug response (IC50) data from cancer cell lines to predict sensitivity for the drugs. These models were applied to bulk RNA‐seq profiles from the TCGA and the GTEx project to generate the predicted IC50 values across a wide range of normal and tumor tissues, where lower IC50 values indicate higher predicted sensitivity. We obtained the data from a public portal (https://manticore.niehs.nih.gov/cancerRxTissue) and compared the drug sensitivity between the peak and trough expression windows of the drug‐targeted gene. For each drug–target gene pair, the peak window was defined as ±6 h centered on the peak expression time point of the targeted gene. The trough window was defined as the remaining hours outside the peak window.

### 4.7 Prediction of Upstream Transcriptional Regulators

To identify candidate upstream regulators mediating the reprogramming of rhythmicity, we employed two complementary approaches integrating transcriptomic data with TF binding profiles, H3K27ac ChIA‐PET chromatin interactions, and GRO‐seq data derived rhythmic enhancer activity. Specifically, we focused on genes that commonly lost their rhythmic expression across multiple pathological conditions and examined whether their regulatory regions were bound by specific transcription factors.

In the first approach, TF binding profiles were used to assess enrichment of TF binding near common rhythmicity‐loss genes. We first constructed a comprehensive index of 73 TF binding profiles in mouse liver based on publicly available ChIP‐seq peak datasets. For each gene that exhibited loss of rhythmicity in more than three pathological conditions, we defined a genomic window spanning 2.5 kb upstream to 1 kb downstream of the TSS to capture core promoter activity and proximal *cis*‐regulatory elements. These regions were then queried against the TF binding index using GIGGLE [37], a similarity search algorithm that ranks the statistical significance of overlap between query regions and indexed TF binding sites. Enrichment was quantified using the GIGGLE score. Enrichment was quantified using the GIGGLE score. This approach enabled us to prioritize TFs whose binding profiles were significantly enriched near genes with disrupted rhythmic expression.

In the second approach, we used chromatin interactions detected by H3K27ac ChIA‐PET and rhythmic enhancer activity inferred from eRNA transcription to identify potential upstream regulators. Rhythmic enhancers were identified based on bidirectional transcription signals derived from GRO‐seq data [24, 45]. Candidate regulatory regions were defined by the intersection of rhythmic enhancer peaks and a genomic window spanning 2.5 kb upstream to 1 kb downstream of the TSS of genes that consistently lost rhythmicity across three pathological conditions. To predict transcription factor regulators, we performed motif enrichment analysis on these candidate regions using HOMER [72].

In both approaches, transcription factors were ranked based on enrichment scores, and the top overlapping candidates were considered putative upstream regulators, with higher enrichment scores indicating greater confidence in TF–target associations.

### 4.8 Time‐of‐Day‐Dependent Estrogen Treatment

Mice were housed under controlled environmental conditions (22 ± 2°C; 40%–60% humidity) on a 12‐h light/12‐h dark cycle (lights on at 6:00 a.m. [Zeitgeber Time 0, ZT0]; lights off at 6:00 p.m. [ZT12]). Animals had ad libitum access to standard chow (LabDiet 5001, PMI Nutrition International) and water. Before and after OVX surgery and throughout the E2 treatment period, mice were group‐housed in cages of 4, with treatment groups mixed within cages (2 E2‐treated and 2 vehicle‐treated mice per cage) to minimize cage effects. At 28 weeks of age, twenty female mice underwent bilateral ovariectomy. After a 3‐week recovery period, the mice were randomly assigned to receive either 17β‐estradiol (E2; MedChemExpress, HY‐B0141) or vehicle control via oral gavage, administered daily at either ZT4 (corresponding to the trough of ERα expression) or ZT16 (corresponding to its peak) for four weeks (*n* = 5 per treatment group). E2 was first dissolved in ethanol at 1 mg/mL, then freshly diluted 1:1000 in sterile saline prior to each administration, yielding a final solution containing 0.01% ethanol. Mice in the E2‐treated groups received 100 µg/kg of E2 in 0.01% ethanol‐saline via oral gavage. Vehicle‐treated mice received an equivalent volume of 0.01% ethanol in sterile saline. All animal experiments were approved by the Institutional Animal Care and Use Committee (IACUC) at Baylor College of Medicine (protocol numbers: AN‐8678 and AN‐6583).

### 4.9 Metabolic Phenotype Measurement

Spontaneous wheel‐running activity and daily food intake were assessed using the PhenoMaster System through the Mouse Metabolism and Phenotyping Core at BCM. A total of 10 E2‐treated mice and 10 vehicle‐treated control mice were transferred from group housing to individual cages equipped with running wheels for continuous monitoring over a four‐day period. The initial 48 h were considered a habituation phase and excluded from analysis to minimize stress‐induced artifacts. During the final two days, spontaneous wheel running of each mouse was recorded at 20‐min intervals under controlled light‐dark conditions. Food intake during this monitoring period was normalized to body weight and reported as daily food consumption (g/g BW/day). These wheel‐running measurements were conducted in the same cohort of mice used for the four‐week E2 treatment experiment, as indicated in Figure 5A. Metabolic data were analyzed using both the CalR software (https://calrapp.org/) and custom R scripts developed in‐house.

Throughout the four‐week treatment period, body weight was measured weekly to monitor treatment‐related changes over time. Full longitudinal body weight data are reported in Table S2; no significant differences between experimental groups were observed prior to treatment initiation. At the end of the treatment, body composition, including fat mass and lean mass, was assessed using quantitative nuclear magnetic resonance (qNMR) with the EchoMRI Body Composition Analyzer.

### 4.10 Triglycerides Measurement

Serum triglyceride levels were quantified using the Triglyceride Liquid Reagent for Diagnostic Set (SB2100430, Stanbio Laboratory). Serum samples were diluted 1:5 in cell lysis buffer (50 mm Tris‐HCl, pH 7.4; 140 mm NaCl; 0.1% TX‐100) to ensure signal linearity. In a 96‐well microplate, 20 µL of each diluted sample or standard was combined with 20 µL of 1% deoxycholate to solubilize lipids, and the plate was incubated at 37°C for 10 min. Then, 200 µL of activated triglyceride reagent was added to each well, and the plate was incubated at 37°C for an additional 20 min. Absorbance was measured at 500 nm using a microplate reader. Triglyceride concentrations were determined by comparison to a standard curve prepared in parallel using known concentrations of glycerol standards provided with the kit.

### 4.11 RNA Extraction and Quantitative PCR

Total RNA was extracted from frozen liver tissue using TRIzol reagent, followed by purification with the RNeasy Mini Kit. 1 µg of total RNA was reverse‐transcribed into complementary DNA (cDNA) in a 20 µL reaction using the High‐Capacity cDNA Reverse Transcription Kit following the manufacturer's instructions. Quantitative real‐time PCR (qPCR) was carried out using SYBR Green PCR Master Mix on a QuantStudio 6 Flex Real‐Time PCR System. The expression levels of target genes were quantified using the standard curve method and normalized to the housekeeping gene *Arbp*. The sequences of the primers are as follows: *Insig2*: forward 5'‐TCATGACACAGTCGTTGGTCC‐3'; reverse 5'‐AACCCAGGTGTCCACAGGTA‐3'. *Map3k13*: forward 5'‐GGGCTGTCTGACAAGGAGTG‐3'; reverse 5'‐CAAACTGCACAGGGTTCTCG‐3'. *Ptgds*: forward 5'‐CACAGAGGAGGACATTGTTTTCC‐3'; reverse 5'‐ACTGACTTCTCTCACCTGCGTTT‐3'.

### 4.12 Western Blot Analysis

Liver tissues were collected from mice treated with vehicle control or E2 during the daytime or nighttime. Protein lysates were prepared using RIPA lysis buffer supplemented with protease and phosphatase inhibitors. Equal amounts of protein were separated by 10% SDS‐PAGE and transferred to 0.2 µm PVDF membranes. As a positive control for insulin signaling activation, liver lysate from mice subjected to 16‐h fasting followed by 45‐min refeeding was included on each blot; this condition robustly activates hepatic insulin signaling through physiological nutrient‐induced insulin secretion [73, 74]. Membranes were probed with the following primary antibodies: total IR (Bioss, bs‐0681R), phospho‐IR (Tyr1355; Bioss, bs‐3492R), total IRS1 (Cell Signaling Technology, 2382S), phospho‐IRS1 (Tyr612; ThermoFisher, 44–816G), total AKT (Cell Signaling Technology, 9272S), phospho‐AKT (Ser473; Cell Signaling Technology, 9271S), and β‐Actin. For each phosphoprotein, the signal intensity was first normalized to β‐Actin to correct for protein loading, and then expressed as a ratio to its respective total protein level. Uncropped blot images are provided in Figure S6.

### 4.13 Web Portal Development

The web portal was developed using the Python Flask framework, deployed behind a Nginx HTTP server, and hosted on the Jetstream2 cloud platform. A RESTful API layer built with Flask enables programmatic access to search queries, rhythmicity evaluation, dynamic figure rendering, and batch data submission. The web interface was implemented using HTML/CSS, Bootstrap 5, and JavaScript, with interactive plots rendered by Plotly.js and R scripts.

The portal offers a user‐friendly entry point to explore rhythmicity across species, tissues, and regulatory layers. Key features include gene‐level visualizations, cross‐layer regulatory queries, batch analysis via file uploads, and customizable analytical options. For user‐submitted data, gene expression datasets uploaded for rhythmicity analysis are processed transiently and are not stored, shared, or reused beyond the requested analysis. These data are not accessible to the curators or developers of the RHINO platform, nor are they incorporated into future versions of RHINO or used for training or refinement of the AI‐assisted chatbot or underlying language models. Data processing is performed in‐memory during each session, and no user‐uploaded data is retained after analysis completion.

A comprehensive user guide is available on the “Documents” page of the website, detailing example queries, supported regulatory layers, species, tissues, and instructions for interpretation. The portal also integrates an interactive rhythm chatbot, combining a user‐friendly web interface with a Flask‐based backend service. The chatbot supports real‐time natural language queries and provides interactive feedback, including rhythmic heatmaps and gene expression plots. Backend APIs handle rhythmicity status retrieval, similar gene suggestion, and plot generation based on precomputed time‐series transcriptomic data organized by species, tissue, and gene symbol.

To support natural language‐based querying, we implemented an intent recognition and task routing system utilizing OpenAI GPT APIs. User inputs were processed to extract gene symbols, tissue contexts, and query categories, including rhythmicity detection, similar gene recommendation, cross‐tissue expression comparison, and user‐defined analyses. To enable multi‐turn interactions, such as generating follow‐up plots and command suggestions after an initial query, a memory buffer was implemented to maintain conversational context. The system supports both predefined function calls and fallback responses generated via large language models. This architecture is designed to support the development of additional analytical functions and the integration of new rhythmic datasets.

### Ethics Statement

All animal experiments were approved by the Institutional Animal Care and Use Committee (IACUC) of Baylor College of Medicine (protocol numbers: AN‐8678 and AN‐6583). Human datasets were obtained from publicly available resources, including TCGA, GTEx, and GEO. These datasets are de‐identified and publicly accessible; therefore, additional institutional ethics approval was not required. Genotype data for GTEx individuals were obtained through dbGaP (phs000424.v8.p2) under approved data access authorization and used in accordance with dbGaP data use policies.

## Author Contributions

Y.C., C.C., D.Z., P.L., S.H., Z.S., L.H., and D.G. conceptualized the study, interpreted the data, and contributed to manuscript writing. The manuscript was revised and approved by all authors. P.L., I.B.X., and Y.C. collected the data. Y.C. and P.L. performed data analysis. D.Z. and Y.C. conducted the OVX, gavage, and Western blot experiments. P.S., D.Z., and Y.C. performed metabolic and body composition measurements. R.E.L. and C.M.L. carried out triglyceride assays and qPCR experiments. C.C. and Y.C. developed the RHINO platform, and Y.L. and C.C. maintained its operation.

## Conflicts of Interest

The authors declare no conflicts of interest.

## Supporting information




**Supporting File**: advs76371‐sup‐0001‐SuppMat.pdf.

## Data Availability

The raw genomic sequencing data from the GTEx project V8 are available in the database of dbGaP with accession number phs000424.v8.p2 [https://www.ncbi.nlm.nih.gov/gap/]. Multi‐layer regulatory datasets were obtained from a variety of public resources, including the GEO, TCGA, GTEx project, CistromeDB, ENCODE, and peer‐reviewed publications. A complete list of data sources and accession identifiers is available at: https://hanlaboratory.com/RHINO/Dataset.html.
