# Supplementary material for: RHINO: An Integrative Multi‐Omics Framework Linking Circadian Physiology to Precision Medicine
Source: Adv Sci (Weinh). 2026 Jul 7:e76371. Online ahead of print. doi: 10.1002/advs.76371 (PMC13339356; doi:10.1002/advs.76371)
Supplement: Supplementary file 1 — Supporting File: advs76371‐sup‐0001‐SuppMat.pdf. [file ADVS-9999-e76371-s001.pdf]

Supplementary Materials for

RHINO: An integrative multi-omics framework linking circadian physiology to precision medicine

Ying Chen, Chengxuan Chen, Dishu Zhou, Panpan Liu, Roberto E López-Valiente, Yuan Liu, Cam Mong La, Isabella Beraldo Xavier, Pradip Saha, Zheng Sun, Leng Han, Dongyin Guan

Corresponding author:

Leng Han, Ph.D. (lenghan@iu.edu)

Dongyin Guan, Ph.D. (dongyin.guan@bcm.edu)

The PDF file includes:

Figs. S1 to S9

Tables S1 and S2

Figure S1

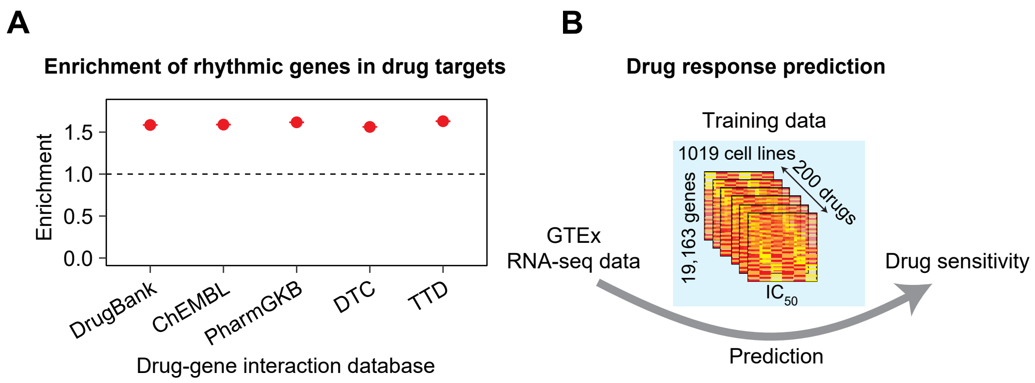

Figure S1. Rhythmic genes are overrepresented among drug targets. (A) Analysis of rhythmic gene enrichment in drug targets. The enrichment is calculated as the ratio of observed rhythmic drug target genes to the expected number. The expected rhythmic gene set was generated by random selection from all expressed genes while preserving expression distributions and detection power. A null model was constructed based on these random samplings, and the median number of drug targets in the expected rhythmic gene sets was used as the expectation. **(B)** Schematic illustrating the prediction of drug responses as described by Li et al.

Figure S2

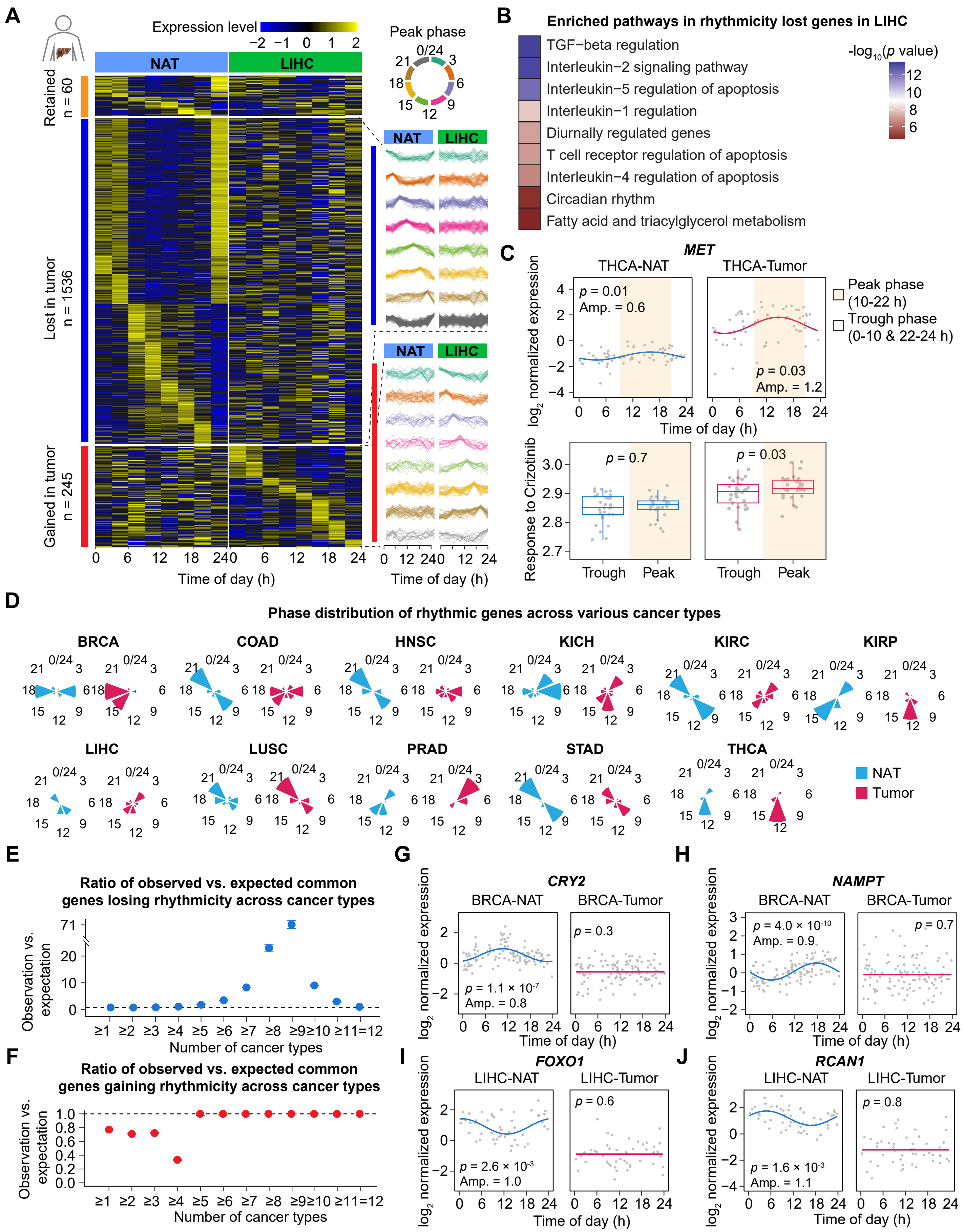

A
Expression level
−2 −1 0 1 2
NAT
LIHC
Retained n = 60
Lost in tumor n = 1536
Gained in tumor n = 245
Peak phase
0/24 3 6 9 12 15 18 21
Time of day (h)
B
Enriched pathways in rhythmicity lost genes in LIHC
TGF−beta regulation
Interleukin−2 signaling pathway
Interleukin−5 regulation of apoptosis
Interleukin−1 regulation
Diurnally regulated genes
T cell receptor regulation of apoptosis
Interleukin−4 regulation of apoptosis
Circadian rhythm
Fatty acid and triacylglycerol metabolism
-log10(p value)
C
MET
THCA-NAT
THCA-Tumor
p = 0.01
Amp. = 0.6
p = 0.03
Amp. = 1.2
Peak phase (10-22 h)
Trough phase (0-10 & 22-24 h)
log2 normalized expression
Time of day (h)
Response to Crizotinib
p = 0.7
p = 0.03
Trough
Peak
D
Phase distribution of rhythmic genes across various cancer types
BRCA
COAD
HNSC
KICH
KIRC
KIRP
LIHC
LUSC
PRAD
STAD
THCA
NAT
Tumor
E
Ratio of observed vs. expected common genes losing rhythmicity across cancer types
Observation vs. expectation
Number of cancer types
≥1 ≥2 ≥3 ≥4 ≥5 ≥6 ≥7 ≥8 ≥9 ≥10 ≥11 =12
F
Ratio of observed vs. expected common genes gaining rhythmicity across cancer types
G
CRY2
BRCA-NAT
BRCA-Tumor
p = 1.1 × 10-7
Amp. = 0.8
p = 0.3
H
NAMPT
p = 4.0 × 10-10
Amp. = 0.9
p = 0.7
I
FOXO1
LIHC-NAT
LIHC-Tumor
p = 2.6 × 10-3
Amp. = 1.0
p = 0.6
J
RCAN1
p = 1.6 × 10-3
Amp. = 1.1
p = 0.8

Figure S2. Tumor-associated circadian remodeling uncovers rhythmic genes relevant to chronotherapy. (A) Heatmap of rhythmic gene expression in human normal liver and liver tumor tissues. **(B)** Pathway enrichment analysis of rhythmicity lost genes in human liver tumor tissue. **(C)** Rhythmic expression of *MET* in matched THCA normal (THCA-NAT) and tumor (THCA-Tumor) samples (top) and predicted responses to Crizotinib in patients with internal circadian phases at the trough versus peak expression phases of *MET* (bottom). **(E-F)** Ratio of observed to expected genes with rhythmicity commonly lost **(E)** or gained **(F)** across multiple cancer types. **(G-J)** Rhythmic expressions of *CRY2* **(G)**, *NAMPT* **(H)**, *FOXO1* **(I)**, and *RCAN1* **(J)** in matched NAT and tumor tissues. These genes display robust rhythmicity in normal tissues but lose oscillation in more than ten cancer types.

Figure. S3

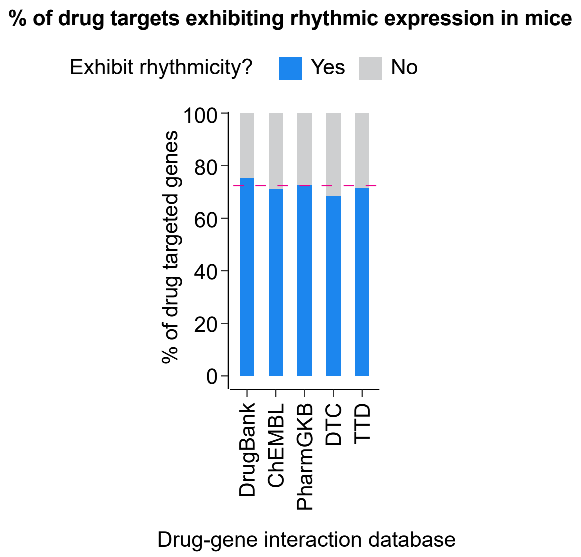

Figure S3. Percentage of drug targets with rhythmic expression in mice. Drug target genes with rhythmic expression are identified based on circadian transcriptomic profiles from various tissues under normal physiological conditions.

Figure S4

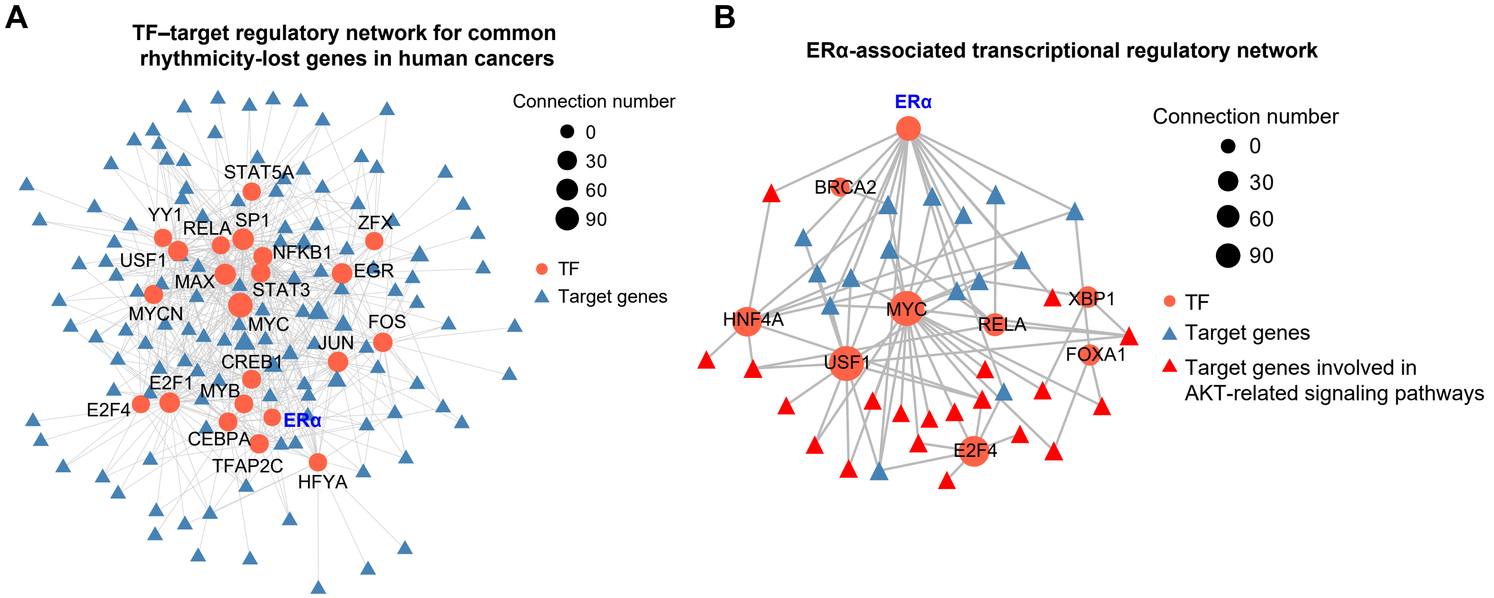

Figure S4. Visualization of TF-target gene regulatory networks. (A) Regulatory network of predicted TFs (orange circles) and their downstream target genes (blue triangles) that lose rhythmicity under more than seven cancer types. Node size is proportional to the number of regulatory connections. **(B)** ERα-associated transcriptional regulatory subnetwork extracted from TF–target network shown in **Fig 4C**. The network shows ERα and its connected transcription factors and target genes. Target genes involved in AKT-related signaling pathways are indicated in red.

Figure S5

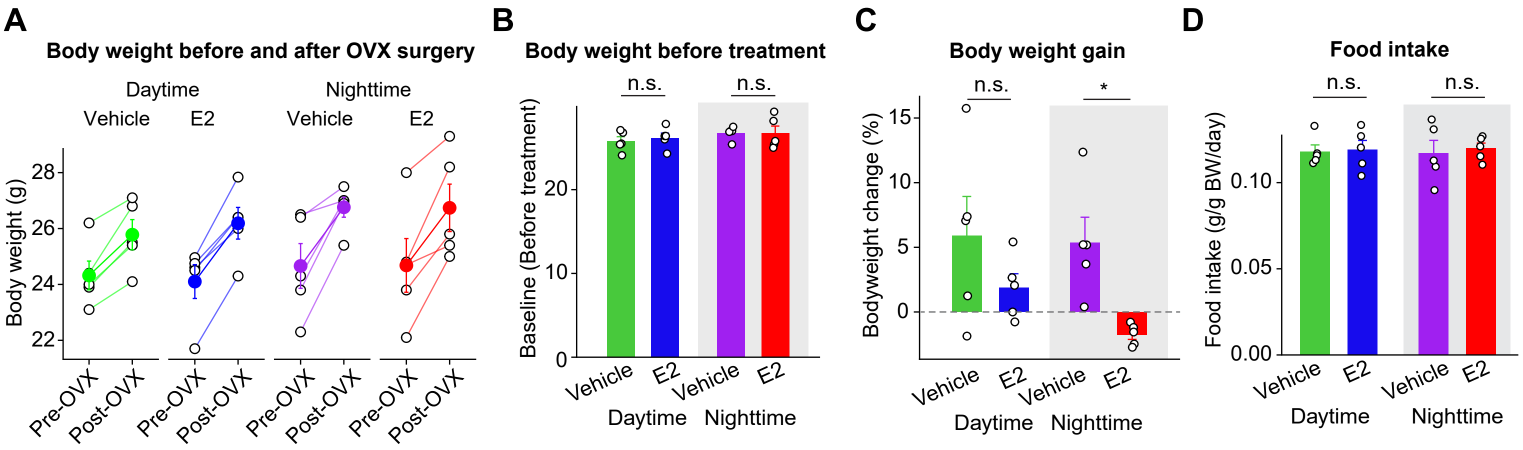

Figure S5. Body weight and food intake measurements across the OVX and E2 treatment timeline. (A) Body weight before and after OVX surgery. Individual paired measurements show body weight at Pre-OVX, assessed before surgery, and Post-OVX, assessed 3 weeks after surgery and serving as the baseline body weight prior to initiation of E2 or vehicle treatment. Connected lines represent individual mice. **(B)** Baseline body weight before E2 or vehicle administration across all four groups. Two-way ANOVA revealed no significant main effect of treatment, time and treatment × time interaction. **(C)** Percentage body weight change over the treatment period relative to baseline. The dashed line at 0% indicates no change from baseline. Two-way ANOVA revealed a significant main effect of E2 treatment (p = 0.009), with no significant main effect of time and treatment × time interaction. **(D)** Daily food intake normalized to body weight (g/g BW/day), measured during the wheel-running monitoring period. Two-way ANOVA revealed no significant main effect of treatment, time and interaction. For all bar graph panels, asterisks denote pairwise comparisons between indicated groups assessed by Wilcoxon rank-sum tests. Data are presented as mean ± SEM. n = 5 mice per group. *p < 0.05, n.s., not significant.

Figure S6

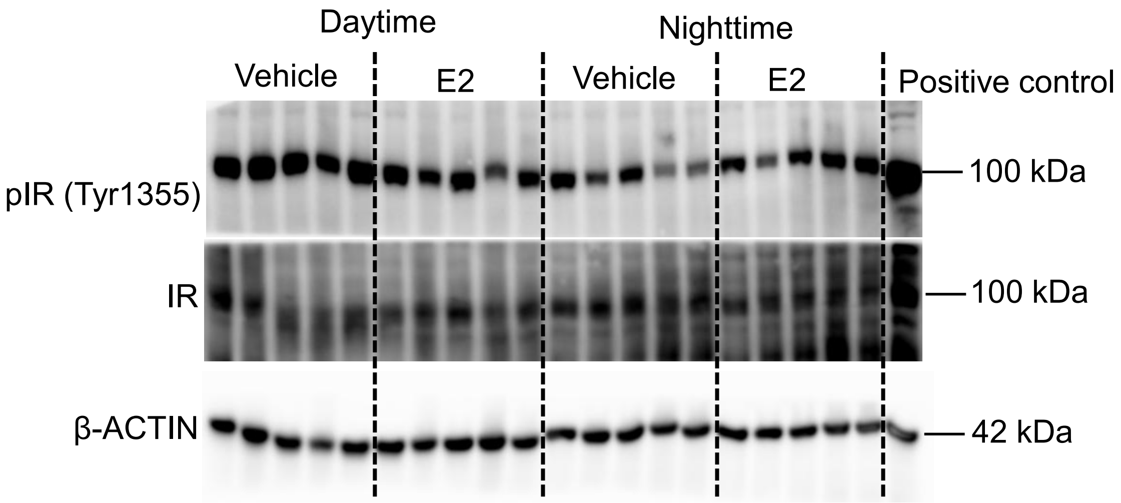

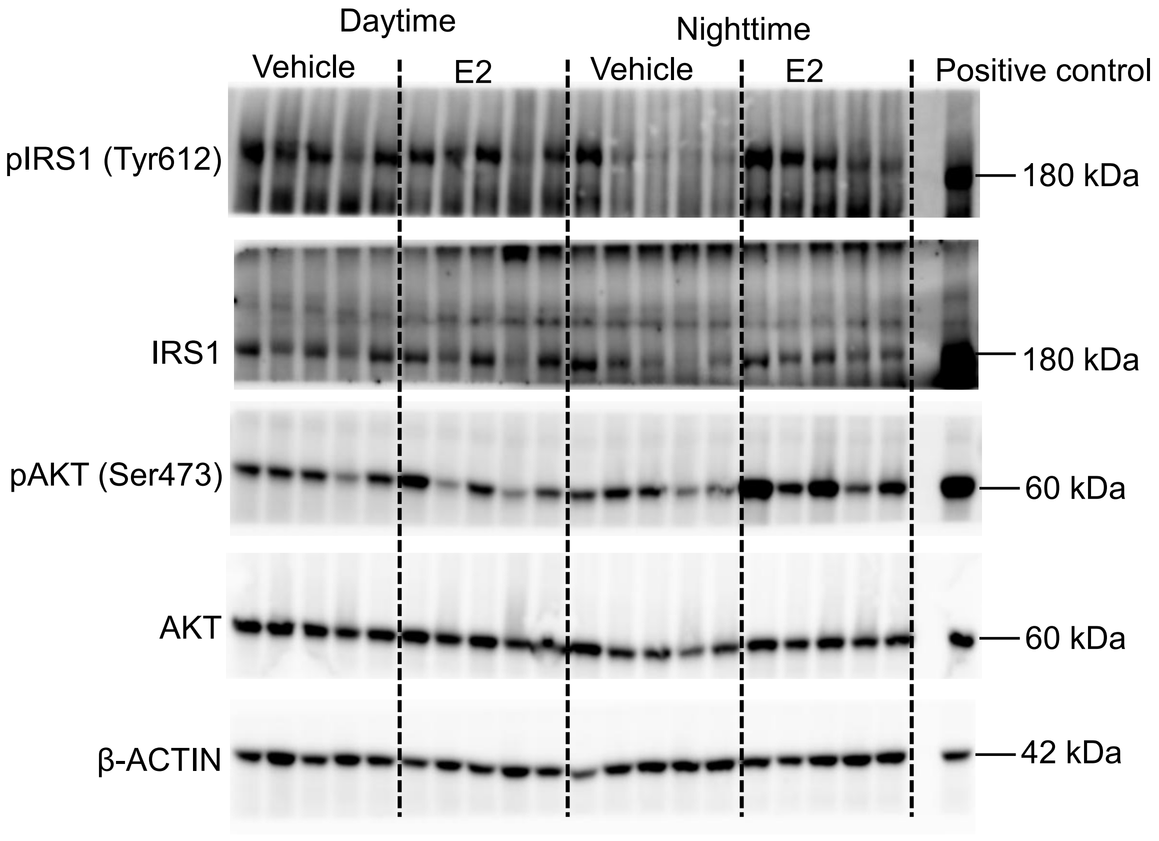

Figure S6. Uncropped Western blot images correspond to Figure 5J. The positive control lane contains liver lysate from mice subjected to 16-hour fasting followed by 45-minute refeeding, which robustly activates hepatic insulin signaling. Molecular weights are indicated on the right.

Figure S7

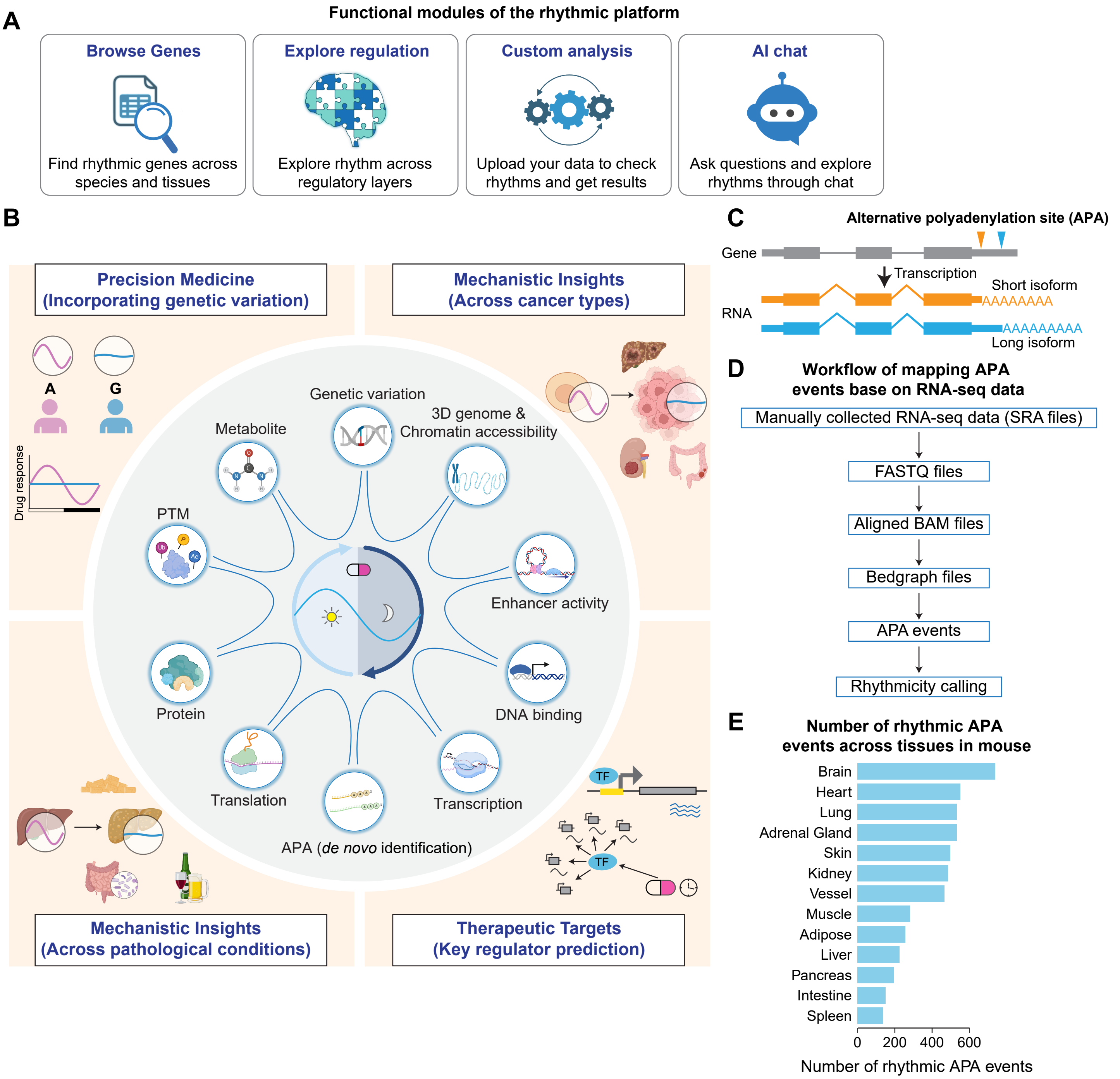

A
Functional modules of the rhythmic platform
Browse Genes
Find rhythmic genes across species and tissues
Explore regulation
Explore rhythm across regulatory layers
Custom analysis
Upload your data to check rhythms and get results
AI chat
Ask questions and explore rhythms through chat
B
Precision Medicine (Incorporating genetic variation)
Mechanistic Insights (Across cancer types)
Mechanistic Insights (Across pathological conditions)
Therapeutic Targets (Key regulator prediction)
A
G
Drug response
Genetic variation
3D genome & Chromatin accessibility
Metabolite
PTM
Enhancer activity
Protein
DNA binding
Translation
Transcription
APA (de novo identification)
TF
C
Alternative polyadenylation site (APA)
Gene
Transcription
RNA
Short isoform
AAAAAAAA
AAAAAAAAA
Long isoform
D
Workflow of mapping APA events base on RNA-seq data
Manually collected RNA-seq data (SRA files)
FASTQ files
Aligned BAM files
Bedgraph files
APA events
Rhythmicity calling
E
Number of rhythmic APA events across tissues in mouse
Brain
Heart
Lung
Adrenal Gland
Skin
Kidney
Vessel
Muscle
Adipose
Liver
Pancreas
Intestine
Spleen
0
200
400
600
Number of rhythmic APA events

Figure S7. RHINO as an AI-powered multi-omics platform for advancing circadian medicine. **(A)** Functional modules of RHINO. The system integrates four core components for comprehensive analysis of gene rhythmicity. Browse genes enable users to query individual genes and examine their rhythmic expression across different species and tissues. Explore regulation supports multi-layer regulatory analysis. Custom analysis allows users to upload gene lists or expression data for rhythmicity detection. AI chat is a natural language interface that enables users to ask questions and interactively explore circadian regulations. **(B)** Overview of the rhythmic regulation across 10 regulatory layers covering 18 data types, including genetic variation, chromatin conformation (Hi-C, 4C, and ChIA-PET), nascent transcription (GRO-seq), ChIP-seq for histone marks and transcription factors, transcriptomics (microarrays and RNA-seq), translation (Ribo-seq), proteomics (mass spectrometry-based), post-translational modifications (phosphorylation, acetylation, succinylation, glycosylation, ubiquitination), and metabolomics and lipidomics. These multi-omic layers reveal comprehensive temporal dynamics in gene regulation. The surrounding quadrants illustrate key applications of RHINO. **(C)** Schematic illustration of the APA event. A gene can produce multiple transcript isoforms by utilizing different polyA sites, resulting in short and long isoforms with distinct 3' ends. **(D)** Workflow for mapping APA events and detecting rhythmicity based on RNA-seq data. Publicly available RNA-seq datasets were manually collected and processed to map the rhythmic APA events. **(E)** Number of rhythmic APA events detected across various tissues in mouse.

Figure S8

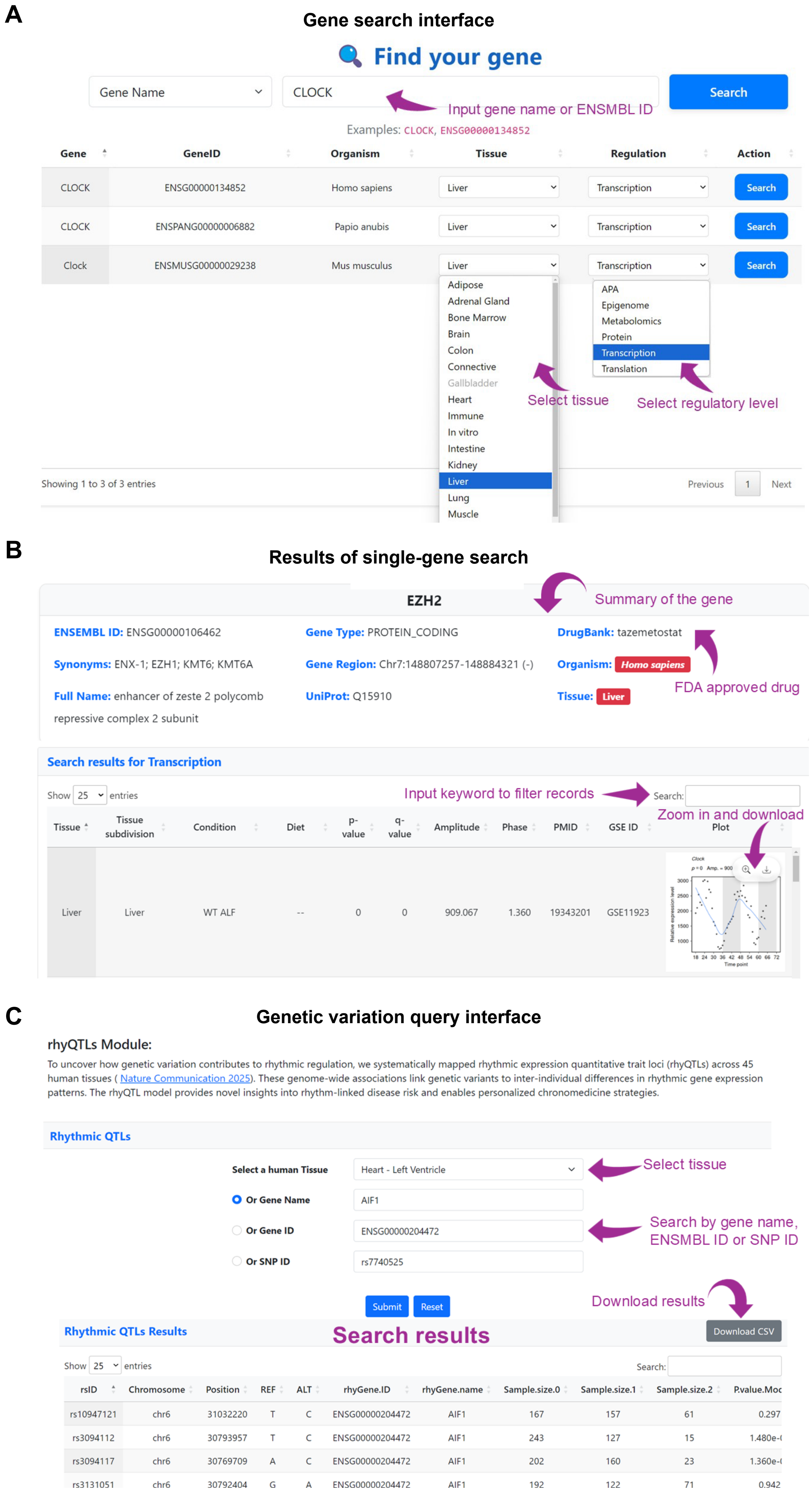
A
Gene search interface
Find your gene
Gene Name
CLOCK
Search
Input gene name or ENSMBL ID
Examples: CLOCK, ENSG00000134852
Gene
GeneID
Organism
Tissue
Regulation
Action
CLOCK ENSG00000134852 Homo sapiens Liver Transcription Search
CLOCK ENSPANG00000006882 Papio anubis Liver Transcription Search
Clock ENSMUSG00000029238 Mus musculus Liver Transcription Search
Adipose
Adrenal Gland
Bone Marrow
Brain
Colon
Connective
Gallbladder
Heart
Immune
In vitro
Intestine
Kidney
Liver
Lung
Muscle
APA
Epigenome
Metabolomics
Protein
Transcription
Translation
Select tissue
Select regulatory level
Showing 1 to 3 of 3 entries
Previous
1
Next
B
Results of single-gene search
EZH2
Summary of the gene
ENSEMBL ID: ENSG00000106462
Gene Type: PROTEIN_CODING
DrugBank: tazemetostat
Synonyms: ENX-1; EZH1; KMT6; KMT6A
Gene Region: Chr7:148807257-148884321 (-)
Organism: Homo sapiens
FDA approved drug
Full Name: enhancer of zeste 2 polycomb repressive complex 2 subunit
UniProt: Q15910
Tissue: Liver
Search results for Transcription
Show 25 entries
Input keyword to filter records
Search:
Zoom in and download
Tissue
Tissue subdivision
Condition
Diet
p-value
q-value
Amplitude
Phase
PMID
GSE ID
Plot
Liver Liver WT ALF -- 0 0 909.067 1.360 19343201 GSE11923
C
Genetic variation query interface
rhyQTLs Module:
To uncover how genetic variation contributes to rhythmic regulation, we systematically mapped rhythmic expression quantitative trait loci (rhyQTLs) across 45 human tissues (Nature Communication 2025). These genome-wide associations link genetic variants to inter-individual differences in rhythmic gene expression patterns. The rhyQTL model provides novel insights into rhythm-linked disease risk and enables personalized chronomedicine strategies.
Rhythmic QTLs
Select a human Tissue
Heart - Left Ventricle
Select tissue
Or Gene Name
AIF1
Or Gene ID
ENSG00000204472
Search by gene name, ENSMBL ID or SNP ID
Or SNP ID
rs7740525
Submit
Reset
Download results
Rhythmic QTLs Results
Search results
Download CSV
Show 25 entries
Search:
rsID Chromosome Position REF ALT rhyGene.ID rhyGene.name Sample.size.0 Sample.size.1 Sample.size.2 P.value.Moc
rs10947121 chr6 31032220 T C ENSG00000204472 AIF1 167 157 61 0.297
rs3094112 chr6 30793957 T C ENSG00000204472 AIF1 243 127 15 1.480e-
rs3094117 chr6 30769709 A C ENSG00000204472 AIF1 202 160 23 1.360e-
rs3131051 chr6 30792404 G A ENSG00000204472 AIF1 192 122 71 0.942

Figure S8. Overview of query interfaces in the RHINO platform. (A) Gene search interface. Users can search for a gene of interest by name or ENSEMBL ID, and select the organism, tissue, and regulatory layer (e.g., transcription, translation, APA, etc.) to explore its rhythmicity. **(B)** Results of single-gene search. The output includes a gene summary and rhythmicity parameters such as amplitude, phase, and statistical significance, along with visualizations. **(C)** Genetic variation query interface. Users can search for genetic variations associated with rhythmic expression by gene name, ENSEMBL ID, or SNP ID across human tissues, and download the corresponding results.

Figure S9

A

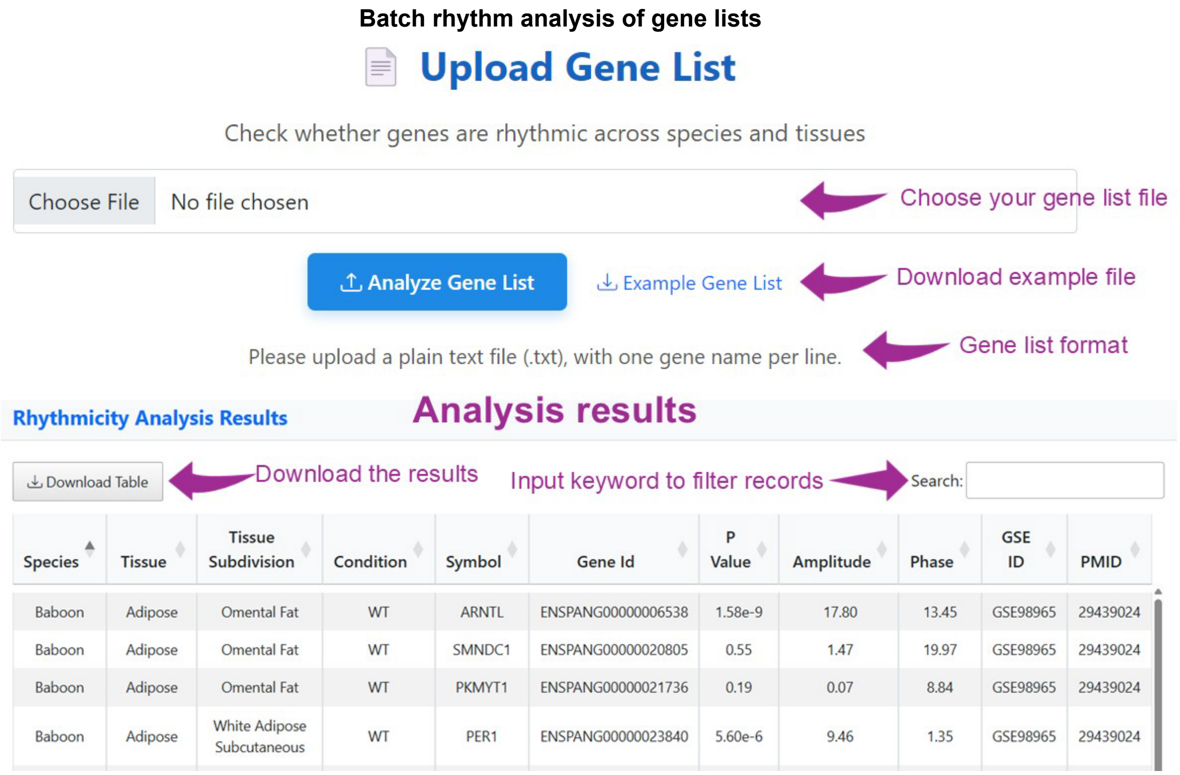

Batch rhythm analysis of gene lists
Upload Gene List
Check whether genes are rhythmic across species and tissues
Choose File
No file chosen
Choose your gene list file
Analyze Gene List
Example Gene List
Download example file
Please upload a plain text file (.txt), with one gene name per line.
Gene list format
Rhythmicity Analysis Results
Analysis results
Download Table
Download the results
Input keyword to filter records
Search:
Species | Tissue | Tissue Subdivision | Condition | Symbol | Gene Id | P Value | Amplitude | Phase | GSE ID | PMID
Baboon | Adipose | Omental Fat | WT | ARNTL | ENSPANG00000006538 | 1.58e-9 | 17.80 | 13.45 | GSE98965 | 29439024
Baboon | Adipose | Omental Fat | WT | SMNDC1 | ENSPANG00000020805 | 0.55 | 1.47 | 19.97 | GSE98965 | 29439024
Baboon | Adipose | Omental Fat | WT | PKMYT1 | ENSPANG00000021736 | 0.19 | 0.07 | 8.84 | GSE98965 | 29439024
Baboon | Adipose | White Adipose Subcutaneous | WT | PER1 | ENSPANG00000023840 | 5.60e-6 | 9.46 | 1.35 | GSE98965 | 29439024

B

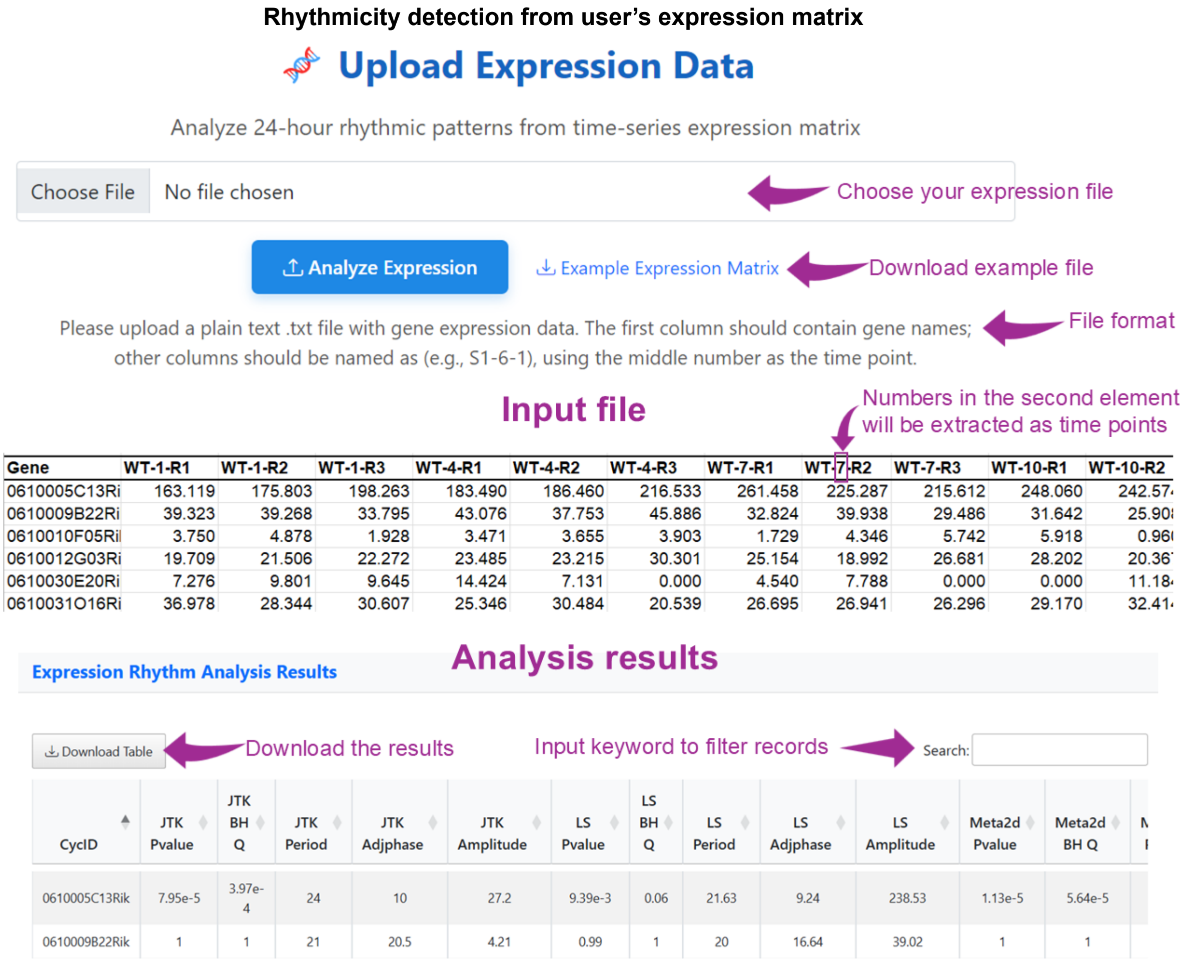

Rhythmicity detection from user's expression matrix
Upload Expression Data
Analyze 24-hour rhythmic patterns from time-series expression matrix
Choose File
No file chosen
Choose your expression file
Analyze Expression
Example Expression Matrix
Download example file
Please upload a plain text .txt file with gene expression data. The first column should contain gene names;
other columns should be named as (e.g., S1-6-1), using the middle number as the time point.
File format
Input file
Numbers in the second element will be extracted as time points
Gene | WT-1-R1 | WT-1-R2 | WT-1-R3 | WT-4-R1 | WT-4-R2 | WT-4-R3 | WT-7-R1 | WT-7-R2 | WT-7-R3 | WT-10-R1 | WT-10-R2
0610005C13Ri | 163.119 | 175.803 | 198.263 | 183.490 | 186.460 | 216.533 | 261.458 | 225.287 | 215.612 | 248.060 | 242.57
0610009B22Ri | 39.323 | 39.268 | 33.795 | 43.076 | 37.753 | 45.886 | 32.824 | 39.938 | 29.486 | 31.642 | 25.90
0610010F05Ril | 3.750 | 4.878 | 1.928 | 3.471 | 3.655 | 3.903 | 1.729 | 4.346 | 5.742 | 5.918 | 0.96
0610012G03Ri | 19.709 | 21.506 | 22.272 | 23.485 | 23.215 | 30.301 | 25.154 | 18.992 | 26.681 | 28.202 | 20.36
0610030E20Ri | 7.276 | 9.801 | 9.645 | 14.424 | 7.131 | 0.000 | 4.540 | 7.788 | 0.000 | 0.000 | 11.18
0610031O16Ri | 36.978 | 28.344 | 30.607 | 25.346 | 30.484 | 20.539 | 26.695 | 26.941 | 26.296 | 29.170 | 32.41
Expression Rhythm Analysis Results
Analysis results
Download Table
Download the results
Input keyword to filter records
Search:
CycID | JTK Pvalue | JTK BH Q | JTK Period | JTK Adjphase | JTK Amplitude | LS Pvalue | LS BH Q | LS Period | LS Adjphase | LS Amplitude | Meta2d Pvalue | Meta2d BH Q
0610005C13Rik | 7.95e-5 | 3.97e-4 | 24 | 10 | 27.2 | 9.39e-3 | 0.06 | 21.63 | 9.24 | 238.53 | 1.13e-5 | 5.64e-5
0610009B22Rik | 1 | 1 | 21 | 20.5 | 4.21 | 0.99 | 1 | 20 | 16.64 | 39.02 | 1 | 1

Figure S9. Web interfaces for user-submitted rhythmicity analysis. (A) Batch rhythm analysis of gene lists. Users can upload a list of gene names to assess whether these genes exhibit rhythmic expression across species, tissues, and conditions. The output includes rhythmicity statistics such as p value, amplitude, and phase. **(B)** Rhythmicity detection from user-submitted expression matrices. Users can upload time-series gene expression data to identify 24-hour rhythmic patterns, allowing direct evaluation of rhythmicity in their own datasets.

Table S1. rhythmic genes in LIHC-NAT in translation and metabolism related pathways.

Symbol	Gene	*p* value	Phase (0-24 h)	Amplitude	Pathway annotation
ARFGAP3	ENSG00000242247	1.76E-06	23.74916	0.852651	Translation
ATG2A	ENSG00000110046	0.001062	23.54033	0.643159	Translation
BRIX1	ENSG00000113460	3.88E-05	23.67487	0.739129	Translation
DNAJC2	ENSG00000105821	0.00012	23.28732	0.618471	Translation
GOLPH3	ENSG00000113384	2.71E-09	23.88095	1.245464	Translation
IPPK	ENSG00000127080	5.67E-08	23.03845	0.758664	Translation
MAK16	ENSG00000198042	9.19E-07	23.84132	0.73083	Translation
MPHOSPH10	ENSG00000124383	0.006077	23.01708	0.589174	Translation
MT1A	ENSG00000205362	0.000229	22.7505	2.165334	Translation
NIFK	ENSG00000155438	2.13E-06	23.82315	0.899847	Translation
RNF6	ENSG00000127870	5.66E-05	23.77418	0.591856	Translation
RPS28	ENSG00000233927	0.013132	17.31432	1.073139	Translation
TRAPPC14	ENSG00000146826	0.001752	23.50726	0.58757	Translation
UBE2J1	ENSG00000198833	1.63E-07	23.91525	0.616971	Translation
VCPIP1	ENSG00000175073	0.000198	23.78142	0.641383	Translation
YTHDF2	ENSG00000198492	1.81E-07	23.67967	0.615636	Translation
ZC3H13	ENSG00000123200	0.000797	23.25827	0.631484	Translation
ADH1A	ENSG00000187758	0.036972	9.254362	0.66352	Metabolism
CNDP1	ENSG00000150656	0.023189	5.402337	1.618496	Metabolism
COQ10B	ENSG00000115520	1.28E-07	0.616618	0.768719	Metabolism
CYP2C18	ENSG00000108242	0.023782	9.539818	0.655394	Metabolism
CYP7A1	ENSG00000167910	0.037561	9.146835	1.94727	Metabolism
DHODH	ENSG00000102967	0.000424	0.457899	1.175141	Metabolism
FDPS	ENSG00000160752	0.001741	9.199615	1.126672	Metabolism
FGF21	ENSG00000105550	0.011424	9.483393	2.082428	Metabolism
GCLM	ENSG00000023909	0.000362	1.878798	0.919424	Metabolism
GSTT2	ENSG00000099984	0.047713	1.255591	1.949362	Metabolism
GYG1	ENSG00000163754	1.47E-05	0.459231	0.690447	Metabolism
HSD17B13	ENSG00000170509	0.035256	8.744882	0.682333	Metabolism
MOGAT2	ENSG00000166391	0.02289	11.56425	1.144306	Metabolism
NFE2L2	ENSG00000116044	0.00022	0.872625	0.604425	Metabolism
PGM2	ENSG00000169299	3.99E-05	2.615461	1.222926	Metabolism
SIRT1	ENSG00000096717	0.004418	5.669116	0.580436	Metabolism
SLC22A25	ENSG00000196600	0.044135	11.97271	0.711133	Metabolism
SLC38A3	ENSG00000188338	0.000504	9.87257	0.798954	Metabolism
SOAT1	ENSG00000057252	5.40E-06	0.23262	0.646412	Metabolism

Table S2. Body weight of mice throughout the experimental period

Group	Pre-OVX	Post-OVX Recovery (3 weeks)			Treatment (4 weeks)			
	Week0	Week1	Week2	Week3 (baseline)	Week1	Week2	Week3	Week4
Daytime Vehicle	24.3 ± 0.5	23.9 ± 0.5	25.7 ± 0.5	25.8 ± 0.5	25.1 ± 0.6	24.9 ± 0.6	26.3 ± 0.7	27.3 ± 0.9
Daytime E2	24.1 ± 0.6	24.2 ± 0.8	25.8 ± 0.6	26.2 ± 0.6	25.5 ± 0.5	26.1 ± 0.5	26.2 ± 0.7	26.7 ± 0.6
Nighttime Vehicle	24.7 ± 0.8	24.6 ± 0.4	26.7 ± 0.2	26.8 ± 0.4	25.8 ± 0.5	25.8 ± 0.6	27.2 ± 0.7	28.2 ± 0.9
Nighttime E2	24.7 ± 1.0	24.6 ± 0.5	26.5 ± 0.6	26.7 ± 0.8	26.0 ± 0.8	25.9 ± 0.8	25.7 ± 0.5	26.3 ± 0.7

Body weight (g) presented as mean ± SEM. n = 5 per group. Post-OVX recovery week 3 represents the baseline body weight immediately prior to treatment initiation.
